# Mechanical annealing and memories in a disordered solid

**DOI:** 10.1126/sciadv.abo1614

**Published:** 2022-10-05

**Authors:** Nathan C. Keim, Dani Medina

**Affiliations:** ^1^ Department of Physics, Pennsylvania State University, University Park, PA 16802, USA.; ^2^ Department of Physics, California Polytechnic State University, San Luis Obispo, CA 93407, USA.

## Abstract

Shearing a disordered or amorphous solid for many cycles with a constant strain amplitude can anneal it, relaxing a sample to a steady state that encodes a memory of that amplitude. This steady state also features a remarkable stability to amplitude variations that allows one to read the memory. Here, we shed light on both annealing and memory by considering how to mechanically anneal a sample to have as little memory content as possible. In experiments, we show that a “ring-down” protocol reaches a comparable steady state but with no discernible memories and minimal structural anisotropy. We introduce a method to characterize the population of rearrangements within a sample and show how it connects with the response to amplitude variation and the size of annealing steps. These techniques can be generalized to other forms of glassy matter and a wide array of disordered solids, especially those that yield by flowing homogeneously.

## Teaser

The way that deforming a disordered material can relax its structure intersects with the ways the material stores memories.

## INTRODUCTION

**Fig. 1.**
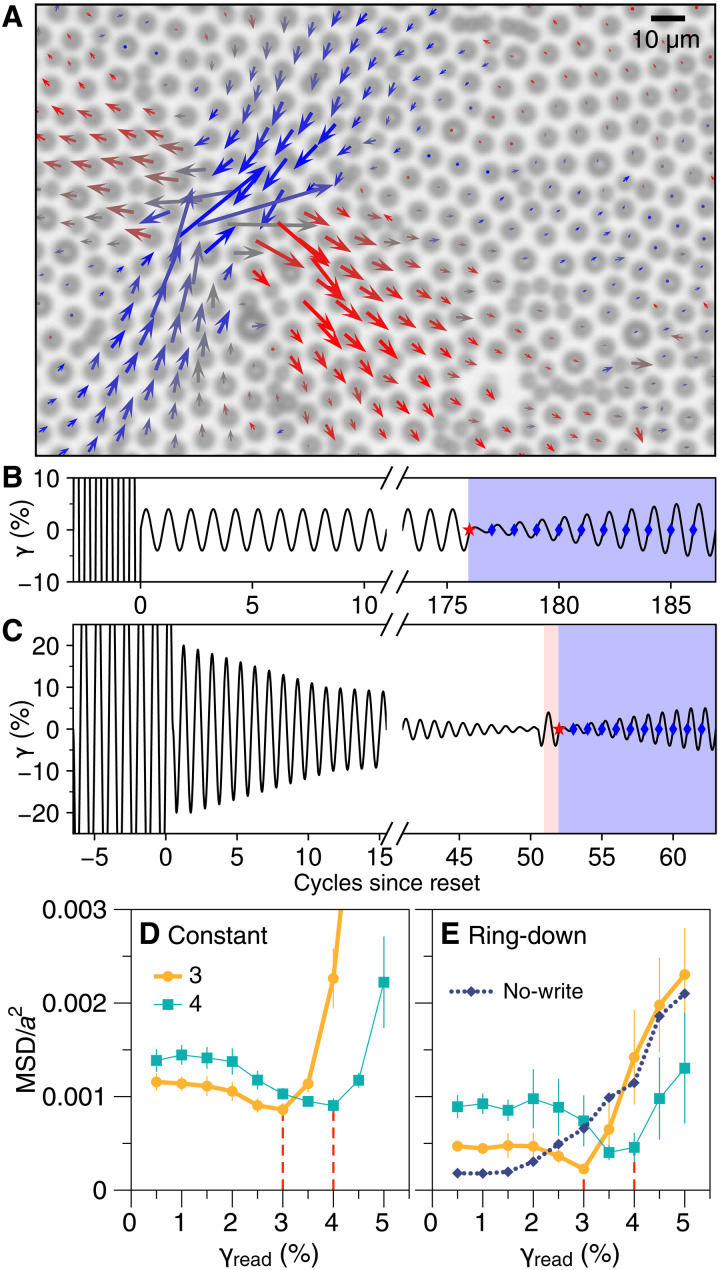
Annealing and single memories. (**A**) 2D disordered solid, made of particles at oil-water interface with electrostatic repulsion. Arrows show displacements (exaggerated 10×) at typical “soft spot” where particles rearrange under horizontal shear. Arrow colors represent direction. (**B**) Constant-amplitude shear strain (γ) protocol: After large-amplitude reset, 176 cycles anneal material and form memory of strain amplitude. Readout (blue shaded region) tests whether the annealed state (red star) can be restored by comparing it with the states after cycles of increasing amplitude γ_read_ (blue dots). (**C**) Ring-down annealing is followed optionally by writing of a memory (red shaded region) and then readout. (**D** and **E**) MSD of particles, normalized by particle spacing *a*, measured during readout as γ_read_ is increased. For each protocol, dips reveal written memories of 3 or 4% by restoring the prereadout states. Vertical dashed lines mark expected memory values. No-write curve in (E) represents no added memories and is possible only with ring-down. Error bars represent SDs of mean for multiple trials. Constant-amplitude data are from experiments in (*26*).

The steps to prepare a solid material for use typically go far beyond forming its chemical constituents into a desired shape. Techniques such as quenching (i.e., rapid cooling) or thermal annealing (slow, staged cooling) and mechanical deformation can markedly affect the final microscopic structure, for example, to increase hardness or strength (*1*). Mechanical methods show particular promise for varying the properties and broadening the applications of amorphous or disordered solids. These materials consist of atoms, particles, drops, or bubbles with negligible long-range order (Fig. 1A), placing them far from any ground state and ensuring a strong dependence on history. For example, despite desirable properties such as corrosion resistance and large strains before failure, bulk metallic glasses tend to start life as brittle materials that fail catastrophically (*2*, *3*) but can be made less or even more brittle through deformation (*4*). Likewise, when studying or using the kinds of foams, concentrated suspensions, or concentrated emulsions found in consumer products or pharmaceuticals, one wishes to erase, or exploit, the effects of prior handling on rheology and microscopic structure (*5*–*7*).

Experiments and simulations that cyclically shear disordered solids have illuminated this mechanical history dependence, aspects of which seem to be common to two-dimensional (2D) and 3D disordered solids with many kinds of microscopic physics (*4*, *8*–*15*). In particular, athermal simulations show that when the material starts in a higher-energy configuration, as if quenched from a high-temperature liquid, and the amplitude of shear is below a critical value, this mechanical annealing lowers the overall energy of the structure (*4*, *9*, *12*, *16*–*22*). Eventually, the material reaches a steady state in which the plastic rearrangements (Fig. 1A) are fully reversible; the rearrangements become periodic, and the particle trajectories become closed loops (*9*, *11*, *13*, *23*, *24*). Since deformation can access a vast set of metastable arrangements of particles in these glassy materials, one might imagine that this precisely periodic behavior can be sustained only by driving at constant amplitude. Reducing the amplitude of shear strain γ_0_ for even one cycle generally leaves the particles in different positions. However, these mechanically annealed solids have an additional, unexpected property: Resuming the previous amplitude restores the positions and the dynamics nearly perfectly (*25*–*28*).

This kind of stability under amplitude variation lets us see that annealing has formed a memory: Without knowing its past, one can probe a sample to reveal the strain amplitude γ*_a_* that was used to anneal it (*14*, *25*, *26*, *29*). We show this in Fig. 1 (B and D) with experiments on the 2D disordered solid from Fig. 1A. We apply a series of cycles with increasing amplitude γ_read_, starting with a small value and ending past γ*_a_* (Fig. 1B). At the end of each readout cycle, the positions of the particles are compared with those at the end of annealing; in Fig. 1D, this is done with the mean squared displacement (MSD), normalized by the typical spacing between particles *a* squared. At small γ_read_, this displacement grows, but it drops near γ_read_ = γ*_a_* when the annealed state of the system is recovered. This observation and others approximate a generic behavior called return point memory (*30*–*32*) that seems to be a property of annealed samples (*26*–*28*). The material’s tendency to retain readable memories, similar to many systems that do not relax to equilibrium (*32*), represents an opportunity for programming, adaptation, and diagnostics but a challenge for annealing. If annealing is meant to relax a material and ultimately lower the entropy of its structure, this outcome would seem to involve as few discernible memories of the past as possible. Could we better understand mechanical annealing if we set out to prepare a blank slate?

In the experiments described here, we prepare a disordered solid with a “ring-down” protocol that gradually decreases the strain amplitude γ_0_ (Fig. 1C), reminiscent of slowly cooling a material from a liquid. As desired, our method leaves no memory of an annealing amplitude, yet we show that it still creates the conditions for return point memory. The memory of an amplitude that was formed by prolonged constant-amplitude annealing turns out to be distinct from return point memories that can be written with single cycles, and here, we disentangle them. We show that another desirable outcome of annealing is to make the material more isotropic and that ring-down annealing does this well, erasing a memory of a shear direction from before annealing. Last, we introduce a more rigorous multicycle test of the response to amplitude variation and show how a model links those results to details of rearrangements obtained in a single cycle of shear. When applied to a finite-size material, this model paradoxically suggests that annealing with oscillatory shear achieves the least memory content by forming every possible memory. Applied more broadly to deformations of thermal glassy matter, our findings point to the value of amplitude variation and memory in annealing, and establish that information from a single cycle of deformation can predict details of the response to amplitude variation.

## RESULTS

The material shown in Fig. 1A is a monolayer of polystyrene particles adsorbed at an oil-water interface, with two diameters to inhibit crystalline order. Because of the particles’ long-range electrostatic repulsion (*33*), each particle is mechanically overconstrained by its neighbors but does not touch them, so that particles form a soft, frictionless jammed 2D solid (see details of samples and apparatus in Materials and Methods). We shear the material at 0.05 Hz in a custom interfacial rheometer (*10*, *11*, *34*–*37*) that lets us synchronously track ∼24,000 particles in a portion of a much larger monolayer. Deformations are approximately quasistatic relative to the time scale of rearrangements. Our analysis is based on shear strain obtained directly from particle positions, γ_aff_, but for clarity, we report the nominal values representing the intended strain of the rheometer (e.g., γ = 3%, −3.5%) unless otherwise noted. We find that γ_aff_/γ ∼ 0.88 because of the stiffness (elastic modulus) of the material (*10*, *11*, *34*, *35*, *38*).

Each experiment begins by shearing with a large strain amplitude of ∼50% for several cycles to “reset” the material and erase memories and prior annealing [e.g., Fig. 1 (B and C)]. The several small, distinctive aggregates and voids in the field of view (see Materials and Methods) are replaced with new ones after this protocol, suggesting that each experiment uses a different sample of particles in the monolayer, which we then anneal. The ring-down annealing protocol (e.g., Fig. 1C) is meant to gradually “cool” the material from plastic flow at the largest strain amplitude (γ_0_ = 20%) to a quiescent elastic solid at the smallest amplitude (γ_0_ = 0.25%). Prior experiments showed that for this system, repeating steady states and memory formation happen at γ_0_ < γ_irreversible_ ∼ 7% (*10*, *11*, *26*, *38*). Therefore, while we begin by decrementing the amplitude γ_0_ in steps of Δγ = 1%, below γ_0_ = 10%, we take smaller steps Δγ = 0.25%, based on the presumption that the material can form memories, so the discreteness of the steps affects the final outcome.

### Effect of annealing on microscopic structure

**Fig. 2.**
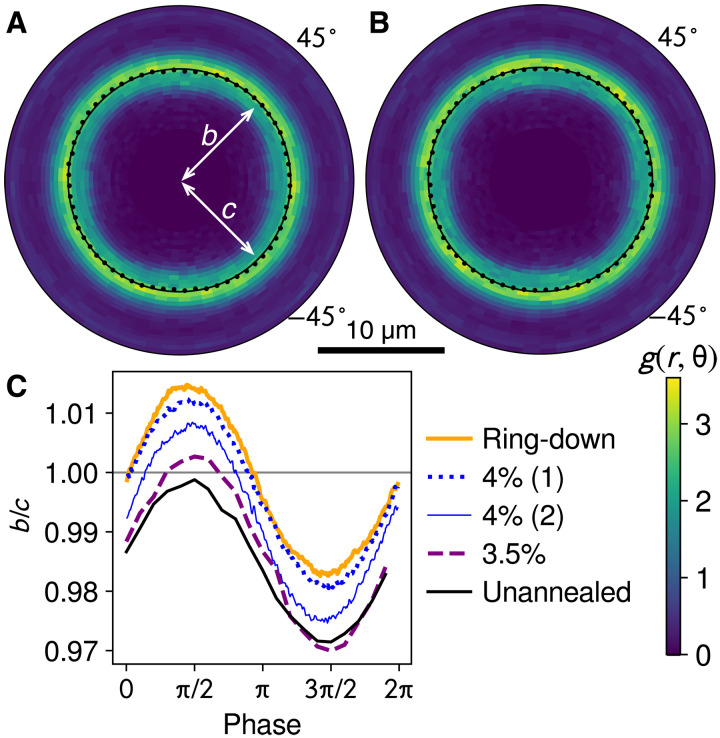
Annealing and microstructure. (**A**) Pair correlation function *g*(*r*, θ) at the end of large-amplitude reset, i.e., unannealed. The color bar is at the lower right of the figure. Black dots indicate nearest-neighbor peak at each θ. The black circle is for comparison, showing that the ring is slightly elongated in the −45^∘^ direction. Ellipse fit to the peaks (not drawn for clarity) has semiaxes *b* and *c*, constrained to the ±45^∘^ directions. (**B**) Corresponding plot after ring-down annealing, showing no single axis of elongation. (**C**) Ellipse axis ratio *b*/*c* during 3.5% amplitude cycles after different annealing, showing that ring-down relaxes underlying asymmetry; constant-amplitude annealing at 4% (two trials) and 3.5% has a smaller, inconsistent effect. The upper three curves are three-frame rolling averages from experiments with more video frames.

Both constant-amplitude and ring-down annealing systematically change the material’s microstructure. Figure 2 shows pair correlation functions *g*(*r*, θ) that characterize the average positions of each particle’s nearest neighbors, where we divide *r* into bins of width at 0.5 pixels and θ into bins of width of 360^∘^/64. From continuum mechanics, we expect that a positive shear strain γ ≪ 1 would extend the microstructure along the θ = 45^∘^ principal axis and compress it along the −45^∘^ axis; an initially circular *g*(*r*, θ) would become an ellipse. To measure this anisotropy, along each azimuthal direction θ, we take the 1D *g*(*r*) and find the center of mass of the first peak with subpixel resolution. We then fit these peak positions with an ellipse that has semiaxes *b* along the 45^∘^ axis and *c* along the −45^∘^ axis.

Intuitively, the condition *b* = *c* should correspond to mechanical equilibrium, with no imposed shear stress in either direction. However, Galloway *et al.* (*39*, *40*) found that an elliptical signature could be detected even at equilibrium and that it survived further shear deformations below the yielding transition, so that its disappearance was a proxy for yielding. They identified it as the memory of an earlier large plastic deformation, consistent with scattering results from bulk metallic glasses that were previously deformed (*4*, *41*, *42*). Teich *et al.* (*43*) showed that the remnant anisotropy was most evident in regions that resisted rearrangements, giving this memory its stubborn persistence up to the point of yielding. These results prompt the question of whether mechanical methods could ever truly erase this anisotropy once it has formed: Large-amplitude shear erases the memory, but might necessarily leave behind another one when it stops.

Our experiments not only observe this persistent memory of a direction but also show that mechanical annealing can remove it. Figure 2A shows that immediately after large-amplitude shear, although the material is ostensibly undeformed (γ = 0), the quenched microstructure is asymmetric with *b*/*c* < 1. On the other hand, the ring-down–annealed sample in Fig. 2B does not show this anisotropy; the peaks are farther along both 45^∘^ and −45^∘^ equally, which is likely an artifact of the camera’s pixel grid. To place these observations in context, Fig. 2C plots the asymmetry *b*/*c* over a cycle of shear with amplitude 3.5%, for an unannealed sample immediately after reset, a sample annealed with constant 3.5% amplitude for 100 cycles, two samples annealed with constant 4% amplitude for 128 cycles, and a sample prepared with ring-down annealing. The 4% annealed and ring-down samples were observed during the readout protocol. The asymmetry oscillates with imposed shear, as expected, but annealing can erase the underlying asymmetry of the quenched microstructure, making the oscillation nearly symmetric about *b*/*c* = 1. Moreover, ring-down annealing does this consistently and not just in the representative curve plotted: Across 13 trials, the mean of *b*/*c* at zero strain, either just after annealing or during readout, is 0.9993 with SD of 0.0024 (histogram in the Supplementary Materials). By contrast, the effectiveness of constant-amplitude annealing seems to depend on the amplitude and may be less consistent: The second trial with 4% amplitude in Fig. 2C has *b*/*c* outside the range of the 13 ring-down trials.

It is likely that one could improve the constant-amplitude results by choosing a larger amplitude, specifically the critical amplitude that simulations suggest leads to statistically identical structures from disparate initial conditions (*20*–*22*). However, those same simulations show that just above that amplitude, the response begins to take on the character of irreversible plastic flow, which is how the memory of a direction was formed in the first place. Ring-down annealing avoids this fine tuning.

### Memories of amplitude

In Fig. 1 (C and E), we show that once a system is prepared by ring-down annealing, a single cycle of shear with amplitude γ_1_ writes a memory of γ_1_. (The cycle is the sequence γ = 0 → γ_1_ → − γ_1_ → 0; the preceding excursion to −γ_1_ is added to smoothly start up shear and ensure a complete cycle that begins and ends at γ = 0.) The form of these readout curves is consistent with the central principle of return point memory (*30*–*32*): As long as the strain is bounded by two turning points (in this case, γ_1_ and −γ_1_), returning the strain to either of those values will restore the system to its state when that strain was last visited. In this case, we expect that once the driving has visited γ_1_ during the readout cycle with γ_read_ = γ_1_, driving the system back to γ = 0 will retrace the same particle motions as during the writing cycle, making the particle positions match those at the end of writing and minimizing the MSD (*26*, *32*, *44*). In the Supplementary Materials, we discuss the variation among individual trials in these experiments and include movies illustrating the 4% readout curve in Fig. 1E. We also show that the same memories are revealed by four other metrics of microscopic change besides MSD.

This kind of writing with a single cycle is seen in other systems with return point memory (*27*, *30*, *44*, *45*), in contrast to the gradual memory formation in systems with multiple transient memories, including non-Brownian suspensions (*44*, *46*, *47*). Prior work on memory readout in disordered solids has written the memories with many repetitions (*14*, *25*, *26*, *29*), conflating the processes of annealing and writing. Our new experiments show that they may be separated. We can also omit the writing step altogether and perform readout immediately after a ring-down, resulting in the monotonically increasing no-write curve in Fig. 1E.

**Fig. 3.**
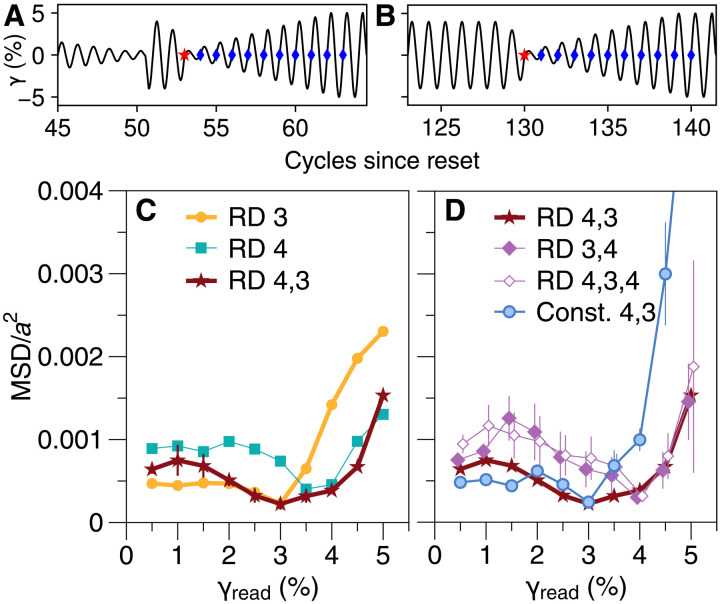
Annealing and nested memories. (**A**) After ring-down, write cycles of 4 and 3% amplitude are applied before readout [marked as in Fig. 1 (B and C)]. (**B**) Alternately, after constant-amplitude annealing at 4%, a single write cycle of 3% is applied before readout. (**C**) Readout with nested amplitudes applied after ring-down (A), labeled RD 4, 3. Single-amplitude curves RD 3 and RD 4 are reproduced from Fig. 1D. The two-amplitude curve is distinct from each single-amplitude curve. (**D**) Results of alternate two-amplitude protocols. RD 4, 3 is from (C). The effect of the smaller amplitude is removed by reversing order of write cycles (RD 3, 4, offset left for clarity) or inserting a final 4% amplitude write cycle (RD 4, 3, 4, offset right). Const. 4, 3 curve is from protocol in (B) and shows clear signatures of two memories: A minimum at 3% due to the single write cycle and a sharp increase in slope past 4% as the limits of prior annealing are exceeded. Error bars for RD 4, 3 and RD 4, 3, 4 show range of two trials; all others show SDs of mean for three trials.

In Fig. 3 (A and C), we show that after ring-down annealing, writing multiple amplitudes has a cumulative effect. We apply a cycle of 4% amplitude and then a cycle of 3%. Because the turning points at ±3% are nested within the previous pair at ±4%, this second cycle does not undo the effects of the first, consistent with the way return point memory may be applied recursively. The resulting “RD 4, 3” curve in Fig. 3C shows that a readout cycle with γ_read_ = 3% amplitude restores the state from the end of writing, as would also be expected with a single memory. However, the subsequent readout above 3% fails to change the system as much as in the single-memory case, revealing that the state of the system is distinct from either single-memory case. We expect that the actual memory at 4% is indicated by an increase in slope of the readout curve (*26*, *32*, *45*), which is difficult to locate with precision in these data. We also verify the hierarchical nature of this memory, consistent with a return point description (*26*, *30*, *31*, *44*): Memories of multiple values must be nested inside each other by writing in descending order and reading in ascending order (the RD 4, 3 curve in Fig. 3D). Reversing the order of writing (“RD 3, 4”) results in a single memory, as does overwriting a dual memory before readout (“RD 4, 3, 4”).

Figure 3D also underscores that while both ring-down annealing (RD 4, 3) and constant-amplitude preparation (Const. 4, 3) allow encoding and readout of the same information, they are not equivalent. Figure 3B shows how we prepare the material with a constant amplitude of 4% and then encode a second memory with a single cycle of 3% amplitude. The greatest difference between these readout curves is for γ_read_ > 3%. Note that unlike in Fig. 1D, the constant-amplitude data in Fig. 3D are from the same set of experiments as the ring-down trials and thus have the same contribution to MSD from errors in particle locations, enabling a quantitative comparison.

We note that for the constant amplitude–annealed samples, readout with amplitude >4% represents the first time that deformations of this magnitude have been performed since the material was quenched from a yielded, flowing regime (the large-amplitude reset protocol). Exceeding the envelope of previous deformations should trigger many additional, latent rearrangements. Work by Mungan *et al.* (*27*) to map out the graph of reachable states under cyclic driving suggests that in general, some of these new rearrangements cannot be reversed by any subsequent deformation; the material is changed permanently. This aspect of the material’s response is reminiscent of the Mullins or Kaiser effects in other systems, where plastic damage constitutes a simple memory of the largest deformation applied (*32*). By contrast, ring-down annealing subjects the quenched material to many cycles with amplitudes ≥4% so that readout with γ_read_ > 4% will trigger few (if any) irreversible rearrangements, consistent with its lower MSD signal.

### Nested memories from ring-down

While we have shown that two memories can coexist in this system, in theory, return point memory allows arbitrarily many values to be stored (*26*, *30*, *31*, *44*). An obvious way to test this prediction is to check whether *n* encoded memories are present in readout data, as we did for *n* = 2 in Fig. 3. Such a readout-based approach is important to a complete description of memory capacity, but it presents the challenge of establishing a method to extract as many features as possible from experimental readout data. One might also write multiple memories in a variety of combinations and test whether each protocol produces a distinctively shaped readout curve, greatly expanding on the approach of Fig. 3C. However, motivated by the ring-down protocol, here, we show that one can much more efficiently test a prerequisite for memory capacity, which we term nested memory resolution: That applying *n* different strain amplitudes in descending order results in *n* distinct states, which can each be recovered later according to return point memory. Our results shed light on the relationship between annealing and memory and on the nature of memory capacity in these materials.

**Fig. 4.**
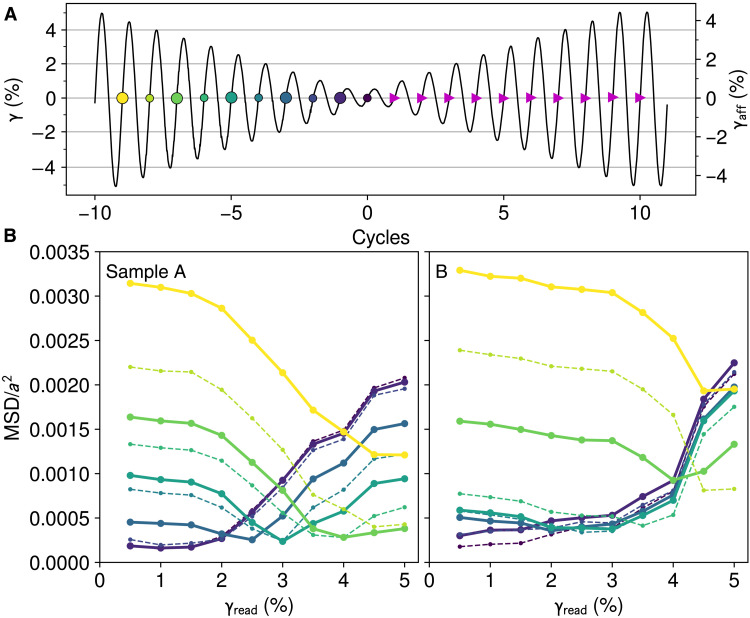
Ring-down as forming nested memories. (**A**) Ring-down annealing (not shown) is followed by 10 more ring-down “write” cycles with decreasing amplitude (ends marked with colored circles) and matching ring-up readout cycles (ends marked with magenta triangles). The left axis shows nominal strain γ used in all plots; the right axis illustrates the relationship to γ_aff_ measured from particle positions. (**B**) Consistent with return point memory, ring-up restores states from ring-down in reverse order. A given ring-down state is compared to each of the ring-up states marked in (A), resulting in one curve in (B). Each curve’s color corresponds to a marker color in (A); to aid distinction, large markers in (A) correspond to solid lines in (B), and small markers correspond to dashed lines. The darkest dashed curve in each plot refers to the end of ring-down; the average of this curve for both samples is in Fig. 1E. Sample A discriminates better among closely spaced memories.

Figure 4A shows our protocol to test nested memory resolution. After ring-down annealing, we apply another ring-down series of 10 cycles with amplitudes from 5% to 0.5%, in steps of 0.5%. At the end of each cycle, the system is ostensibly in a new state that encodes one more memory. Consistent with the hierarchical nature of return point memory, the rearrangements in each cycle are almost completely a subset of those in the previous cycles (fig. S4). As we apply the corresponding ring-up series for readout, return point memory predicts that each of the states from ring-down should be recalled in reverse order. Figure 4B tests this for each of two samples. For example, the 4% curve in each panel (solid green line) compares each state during ring-up to the state after the 4% cycle in ring-down (marked in Fig. 4A with a circle of the same color). If the material had an unlimited ability to resolve memories, we would expect each curve to have a global minimum at the corresponding γ_read_, where the curve would drop sharply to nearly zero MSD. Instead, we see that for sample A in Fig. 4B, the states corresponding to strains ≲2.5% are all similar to each other, suggesting a practical lower bound on distinct memory values. We also see that the 5% readout curve for sample A (solid dark blue) has a large minimum MSD, meaning that this memory state, the first to be recorded and last to be retrieved, was not recalled faithfully and hinting at a practical upper bound on memory values. Between these bounds, the system further struggles to discriminate between 3.5 and 4%, indicating that they are too close to be resolved.

Figure 4B (right) represents the same test performed on sample B, which is the same material, but with a differently randomized structure due to the reset protocols and several other intervening tests. We again see signs of insufficient nested memory resolution, but now, the deficiency is more severe, extending up to γ_read_ ≈ 3%. These two samples show that the ability of the ∼1400 rearranging particles to store and distinguish among strain amplitudes is unexpectedly limited and variable.

### Mapping rearrangement kinematics

To explain the limited resolution revealed in Fig. 4, we return to localized plastic rearrangements similar to the one in Fig. 1A. The loci of these rearrangements are often termed “soft spots,” mesoscopic regions that are predisposed to rearrange under shear, playing the role of shear transformation zones (*8*, *48*, *49*). Experiments by Keim *et al.* (*26*) and simulations by Mungan *et al.* and Regev *et al.* (*27*, *28*) showed how return point memory arises from the hysteresis of many soft spots. Here, we develop a way to efficiently map the kinematic structure of these soft spots before considering how it connects with return point memory. Following earlier analyses, we model each soft spot as a bistable “hysteron” with states +1 and −1 (*11*, *26*, *27*, *48*–*50*). Under forward shear ($\overset{\cdot }{\gamma }>0$), the *i*th soft spot transitions to the +1 state when $\gamma >\gamma_{i}^{+}$; under reverse shear, it transitions to the −1 state when $\gamma <\gamma_{i}^{-}$. The thresholds $\gamma_{i}^{\pm }$ vary among soft spots to represent the disorder of the system. We also require $\gamma_{i}^{+}>\gamma_{i}^{-}$ to represent that each soft spot is dissipative; the rearrangement uses stored elastic energy. This also makes each soft spot hysteretic: When $\gamma_{i}^{-}\leq \gamma \leq \gamma_{i}^{+}$, the state of the *i*th soft spot depends on its history.

Keim *et al.* (*26*) showed previously that this model could explain readout of one or two memories, as in Figs. 1 and 3, if one assumed a uniform distribution of $\gamma_{i}^{\pm }$. Instead, we will measure the actual $\gamma_{i}^{\pm }$ of the many individual soft spots that rearrange and reverse during one cycle of shear with amplitude γ_0_. The simplest method is to start at a chosen time *t*_0_ and measure particle displacements from that instant. However, in practice, this tends to be more sensitive to rearrangements at strains far from γ(*t*_0_), as was evident in a previous measurement (*11*). To construct an unbiased method, we note again that when $\gamma_{i}^{-}<\gamma <\gamma_{i}^{+}$, the *i*th soft spot can be found in either state, depending on the direction of shear. Checking for the direction dependence of each soft spot, at many values of γ during a full cycle in which particle trajectories are closed, will thus reveal the values of $\gamma_{i}^{-}$ and $\gamma_{i}^{+}$.

**Fig. 5.**
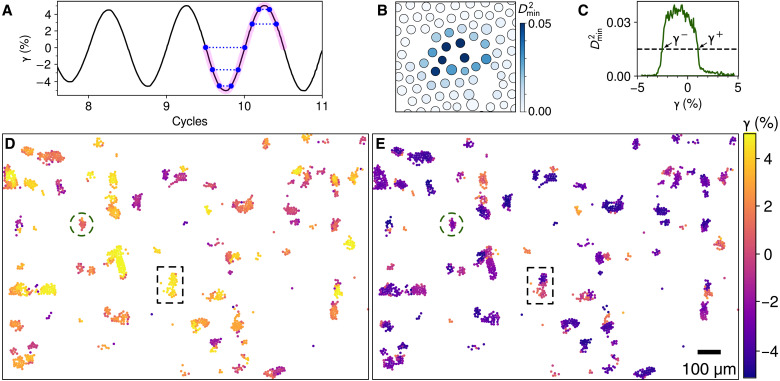
Spatiotemporal structure of rearrangements within a single cycle. (**A**) Sampling method. A cycle with amplitude 5% (highlighted pink), in which particle trajectories are closed, is taken from the end of a readout protocol. Five representative pairs are marked with blue dots and dotted lines, matching each frame during forward shear ($\overset{\cdot }{\gamma }>0$) with the frame at the same γ during reverse shear. (**B**) Squared nonaffine displacement $D_{\text{min}}^{2}$ of particles at the center of the soft spot in Fig. 1A. Voids represent particles that could not be reliably tracked (see text). Fifteen particles shown had values exceeding the threshold 0.015. The largest value is 0.14; the color scale is clipped to show small values clearly. (**C**) $D_{\text{min}}^{2}$ from our sampling method for the particle at the center of the soft spot marked with a circle in (D) and (E). γ^±^ is where signal crosses threshold (dashed line). (**D** and **E**) The top (bottom) panel shows all rearranging particles in the field of view, colored by the strains γ^+^ (γ^−^) at which each rearranges during forward (reverse) horizontal shear. The fixed wall is at the top; the moving needle is at the bottom. The region in the dashed rectangle suggests two strongly coupled soft spots.

In our experiments, we take advantage of the two-cycle segment with γ_0_ = 5% at the end of each readout (e.g., Fig. 4A). We avoid using the very end of this segment, the last frame of the movie, since the strain may not return fully to γ = 0 before video recording ends. We instead select a full cycle from the middle of this segment, highlighted in Fig. 5A. To the extent that the system follows return point memory, this cycle will leave the material unchanged since the immediately preceding visit to γ = 5% already restored the system to the corresponding state during ring-down and erased any smaller memories. Each video frame during forward shear ($\overset{\cdot }{\gamma }>0$) is matched with its counterpart at the same γ during reverse shear. We compute each particle’s squared nonaffine displacement $D_{\text{min}}^{2}$ between this pair of frames (see Materials and Methods), shown in Fig. 5C, and take the particle as being in different states when $D_{\text{min}}^{2}\geq 0.015$, a threshold that we established previously as reliably isolating rearranging regions (*11*, *38*), and that does not have a strong qualitative effect on our results (see fig. S5). We reject any particle that did not have a closed trajectory during the cycle [no particles in the case of Fig. 5 (D and E)] or whose $D_{\text{min}}^{2}$ is elevated for fewer than two frames or for less than half of the frames between its γ^+^ and γ^−^.

Figure 5 (D and E) shows the rearranging particles in sample A, colored by the γ^+^ and γ^−^ that we obtained. Consistent with return point memory, all soft spots returned to their original states at the end of the cycle (sample B had two small exceptions; see fig. S3). We see that neighboring particles tend to have similar values, and there is often very little variation between the core of a small soft spot and its periphery, confirming that the switching of a single soft spot may be treated as a discrete event and that our experiments are approximately quasistatic. We also see that some extended regions that might appear as a single soft spot in a map of, e.g., $D_{\text{min}}^{2}$ actually have two or more distinct values of γ^+^ or γ^−^, indicating that they should be treated as separate hysterons in our model. These locations are opportunities to study small groups of strongly coupled soft spots (*27*, *51*, *52*). In many cases, these interactions are apparent: For example, the region outlined with a dashed rectangle in Fig. 5E rearranges in two stages during reverse shear, with varied γ^−^, but all at once during forward shear, with nearly uniform γ^+^, which is an avalanche behavior identified in the simulations of Mungan *et al.* (*27*) that is explained by a cooperative (i.e., ferromagnetic) coupling.

**Fig. 6.**
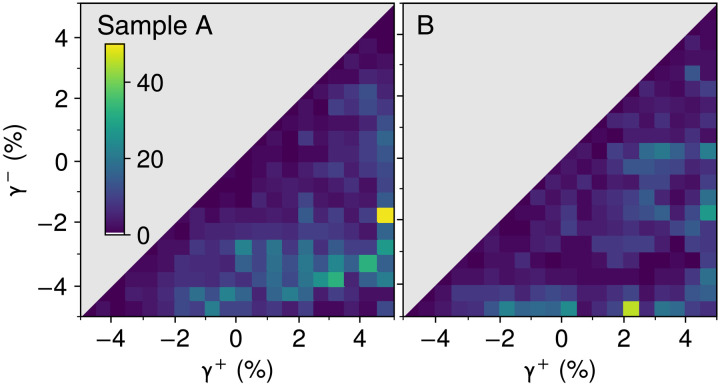
Distribution of soft spot switching thresholds. Histograms of γ^+^, γ^−^ in two identically prepared material samples, A and B. Sample A corresponds to Fig. 5 (D and E). The color indicates the number of particles in each bin.

In Fig. 6, we plot the particles’ γ^+^ and γ^−^ as histograms on the γ^+^ − γ^−^ plane for samples A and B. The bin boundaries of the histograms are the strain amplitudes in our nested memory resolution test. We see large differences in extended portions of these histograms; just as in Fig. 4, we established that memory resolution can vary between samples. We can now consider whether these two kinds of observations are related.

### Predicting response to variable-amplitude shear

**Fig. 7.**
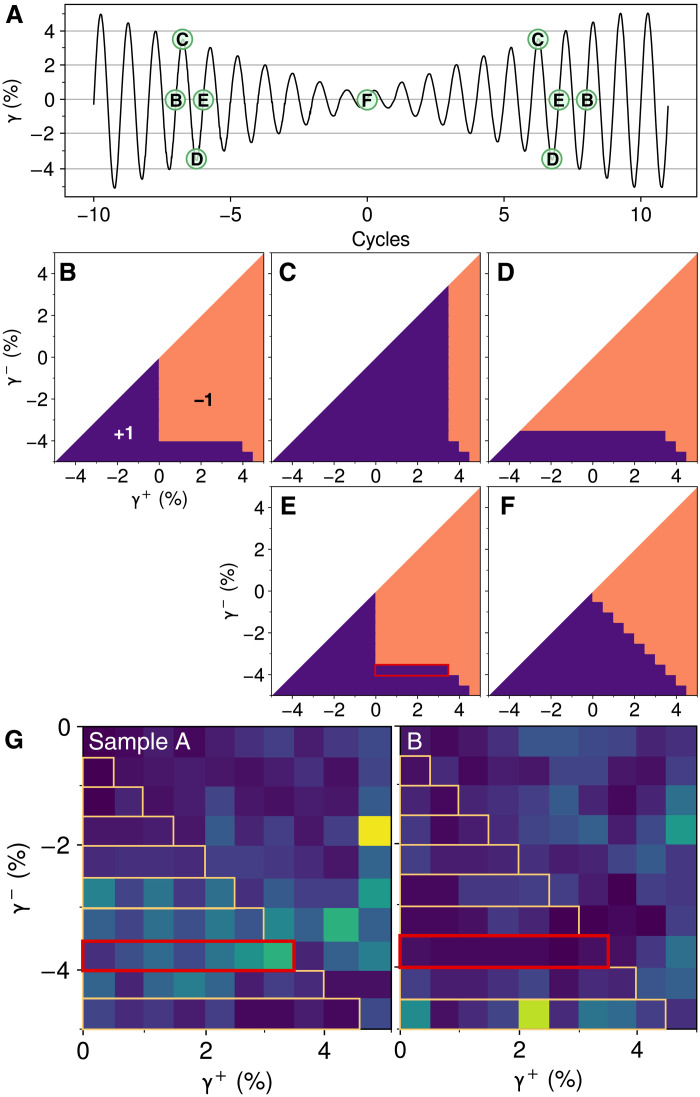
Preisach model of response to amplitude variation. (**A**) Nested memory protocol as in Fig. 4. Labels (B) to (E) mark pairs of identical states, as predicted by return point memory. (**B** to **F**) Diagrams corresponding to labels in (A), showing states of many idealized soft spots, distributed uniformly on the γ^+^ − γ^−^ plane. (B) is at γ = 0 immediately following a cycle with amplitude 4%. To evolve this model to γ = 3.5% at (C) via forward shear, we ensure that every soft spot with γ^+^ ≤ 3.5% has rearranged into its “+1” state, shaded dark purple. Reverse shear to (D) switches every soft spot with γ^−^ ≥ 3.5% to the “−1” state. Returning to γ = 0 at (E) reveals the strip of soft spots with the “+1” state (outlined in red) that distinguish between 4 and 3.5% strain amplitude. Repeating the process with decreasing amplitude leads to (F); increasing amplitude revisits states (B to E). (**G**) Portions of histograms from Fig. 6. The outlined strips show soft spots that let return point memory discriminate between similar strain amplitudes, as tested in Fig. 4; the red outline matches that in (E). Sample B result suggests relatively poor performance below ∼4%, consistent with Fig. 4B.

To predict the response of the soft spots in Fig. 6 to changes in strain amplitude, we adopt the simplifying approximation that soft spots do not influence each other, meaning that the $\gamma_{i}^{\pm }$ are constant. This makes our model of many soft spots equivalent to the model of magnetic hysteresis developed by Preisach (*53*) that is proven to have return point memory (*30*, *31*); our annealing method was inspired by the degaussing method for minimizing the remnant magnetization of a ferromagnet. In Fig. 7 (A to F), we consider how ring-down and ring-up operate on this model. Instead of the experimental histograms in Fig. 6, in Fig. 7 (B to F), we assume that soft spots are continuously distributed on the γ^+^ − γ^−^ plane, and we represent their states. Labels on the strain protocol in Fig. 7A refer to these diagrams. Panels B to E of Fig. 7 are snapshots from one cycle during ring-down. As we shear forward from γ = 0 to γ = 3.5%, we evolve the system from state (B) to state (C) by sweeping rightward on the horizontal axis from 0 to 3.5%, switching soft spots to the +1 state as we go. To complete the cycle, we shear from 3.5 (C) to −3.5% (D) by changing states to −1 as we move downward on the vertical axis, and then, we perform forward shear back to γ = 0 (E). The stair-step pattern in Fig. 7F is the result of the complete sequence of ring-down cycles.

Comparing states (B) and (E), we see that soft spots within one rectangular region on the γ^+^ − γ^−^ plane (outlined in red) are responsible for storing the new memory of 3.5% amplitude and distinguishing it from the retained memory of 4%. No matter how many soft spots are active within a material, if it has none with $0<\gamma_{i}^{+}<3.5\%$ and $-4\%<\gamma_{i}^{-}<3.5\%$, then it cannot resolve this difference; the 3.5% cycle will leave the material unchanged. This lets us interpret the histograms from Fig. 6, portions of which are reproduced in Fig. 7G. We outline and zoom in on the nine regions that encoded the memories from 0.5 to 4.5% in our model. Some regions are relatively deficient in particles, allowing us to predict features of each sample’s response. While both samples appear to have poor susceptibility to nested memories at γ ≲ 2%, sample A should also perform poorly around γ ≃ 4.5%, while sample B should perform poorly around γ ≃ 3%. These features are consistent with the deficiencies that we observed independently in the variable-amplitude tests of Fig. 4, with discrepancies of ±0.5% that are likely due to soft spot interactions and strain nonuniformity (see the Supplementary Materials) that can shift a soft spot into a neighboring histogram bin. Given the magnitude of the variation between samples A and B, limited soft spot populations also partly explain the differences among memory readout curves when repeating trials under identical conditions, as shown by the error bars in Figs. 1D and 3 (C and D).

The success of our Preisach model analysis suggests that although nearby soft spots interact, and despite small strain rate–dependent effects that may nonuniformly shift the timing of rearrangements (see the Supplementary Materials), these effects are perturbative in practice and do not change the broad features of a sample’s response to amplitude variation. We also note that the visible interactions in Fig. 5 (D and E) are cooperative, causing one rearrangement to trigger another at the same γ^+^ or γ^−^, and so are still formally consistent with return point memory (*31*), if not with the Preisach model. Further careful experiments are needed to map out the breakdown of this assumption: As γ_0_ is increased and rearrangements proliferate, these interactions between neighboring soft spots are likely to grow more important, and frustrated interactions (where one rearrangement inhibits a neighboring one) may overwhelm any return point behavior (*51*, *52*, *54*), a transition that could be distinct from the onset of yielding and steady-state irreversibility.

### Memory content and capacity

We can now consider more precisely the relationship between memories of strain and mechanical annealing. We posit that a discernible memory, here, of a past amplitude or direction, is one sign of incomplete annealing. On its face this contradicts our return point memory tests, which suggested that the ring-down protocol with step size Δγ actually writes many nested memories in the material. To resolve this tension, we consider our model of noninteracting soft spots. In an idealized sample with an unlimited number of soft spots, each memory from ring-down would always be distinct from the next, and repeatedly halving Δγ would effectively subdivide each stairstep in Fig. 7F, resulting in an ever-finer structure of nested memories. However, in a finite sample (e.g., Figs. 5 and 6), there may not be enough soft spots to encode these finer amplitude steps. Eventually, decreasing Δγ below some value Δγ_min_ will make no further difference in the outcome of annealing and will not encode any more memories. In this limit, wherein the stairsteps of Fig. 7F are arbitrarily fine, the states of the soft spots would encode only the equilibrium strain γ = 0 around which shearing was symmetric and the fact that a ring-down protocol was used. Instead of a clear stairstep pattern, one would see a line whose roughness was dominated by the discrete nature of the soft spots; the actual written memories would be indistinct in a readout test. While we do not directly establish Δγ_min_ in our experiments, the deficiencies in Figs. 4B and 6 hint that in the context of these ∼24,000-particle samples, it is not much smaller than the Δγ = 0.25% that we already use for annealing. The no-write curve in Fig. 1E, made with Δγ = 0.5%, is approaching a readout with no discernible memories.

Considering Δγ also suggests a way to understand and test memory capacity, the maximum number of memories that can be stored at once. This capacity is saturated in our model when Δγ ≈ Δγ_min_: Inserting a finer step into the series of ring-down amplitudes fails to add a memory, but omitting a step removes a memory and yields a different state, corresponding to a missing stairstep in Fig. 7F. To store many memories, each stairstep must have ≳1 soft spot, and therefore the minimum size of a stairstep should scale with the density of particles on the γ^+^ − γ^−^ plane: $\Delta \gamma_{\text{min}}\propto 1/\sqrt{N}$, where *N* is the number of particles in the system, consistent with the simulation results of Regev *et al.* (*28*). Paradoxically, this interpretation suggests that ring-down uniquely achieves a state with the least memory content by writing as many memories as possible. A system prepared this way will record any subsequent deformation by reverting to an earlier state without the smallest nested memories, a change that can be read out (Fig. 1E).

## DISCUSSION

We have shown that while conventional mechanical annealing with a constant strain amplitude leaves an imprint of that amplitude, applying a decreasing amplitude appears to also anneal the material, while erasing the structural anisotropy from earlier large deformations and leaving no strong signature of a strain. We demonstrated that a key property of our mechanically annealed samples is a kind of stable plasticity, so that to a good approximation, plastic changes in the material can be predictably undone by further deformations. This can be described as return point memory, and our experiments probed it in depth within a ∼8% window of strain (−4 % < γ_aff_ < 4%), first by reading out one or two memories (Figs. 1 and 3) and then by nesting 10 memories together (Fig. 4). The ability to undo plastic deformations seems at odds with the nature of disordered solids: The disordered structure of these materials is not quenched, and there is no guarantee that the soft spots that couple to shear (Fig. 5) will remain from one cycle to the next as we vary the amplitude (*26*, *27*, *48*, *49*). For example, shearing the unannealed material twice with the same amplitude generally yields two different states [e.g., (*9*, *10*)]. Ring-down annealing is exceptional among deformation protocols because it obtains this remarkable stability without leaving any discernible memories. Our findings suggest that future studies of the mechanical annealing process itself, and how it achieves this stability, should also consider amplitude variation.

Recent simulations (*18*, *20*–*22*) that measure a sample’s structural energy stored in interparticle interactions hint at one underlying reason that ring-down can erase the past and achieve this kind of stability. For materials such as ours that yield by flowing homogeneously (rather than with a shear band), ring-down passes through a critical point at the material’s yielding amplitude. Constant-amplitude shear near this critical point erases a sample’s thermal history by approaching a critical energy per particle, the lowest achievable energy for such a material in these athermal studies (*28*, *55*). To obtain the same outcome from ring-down, it is unclear how small the spacing of strain amplitude steps Δγ must be near the critical point, in addition to saturating the memory capacity as discussed above. Simulations have shown that annealing a material to a steady state at a strain amplitude just below this point may require hundreds or even thousands of cycles (*9*, *20*, *56*, *57*), calling for vastly slower annealing than we have demonstrated here. However, these and other studies also show that most of the relaxation takes place early in the process so that a much smaller number of cycles may suffice. Greater efficiency might also result from varying amplitude nonmonotonically near the critical point (*19*). These questions call for further studies over a wide dynamic range of step sizes. Simulations may even show that ring-down can reach lower energies than are possible with any constant amplitude, as in experiments that ramp down driving of a granular packing to approach a structure with the lowest accessible energy (*58*, *59*).

Carefully studying the relationship between annealing and memory offers a clearer view of the complexity of memory behaviors in disordered solids. The multiple memory behavior that was previously observed after many cycles of “training” (*14*, *25*, *26*, *29*), a term carried over from studies of dilute suspensions (*46*, *47*), is actually the superposition of two distinct types of memory: the memory of the envelope of annealing and return point memory within that envelope. This distinction brings to mind examples of how memory behaviors in disparate systems belong to a much smaller set of distinct types, each with characteristic rules for encoding, reading, and erasing (*32*, *44*, *46*). In this case, the envelope of annealing is revealed by the damage that occurs rapidly when that envelope is exceeded (Figs. 1D and 3D) (*60*, *61*), resembling the Mullins or Kaiser effect in other materials, whereby the largest applied deformation is remembered (*32*, *44*). Once annealed, our system allows memories of multiple strains to be written in single cycles, consistent with return point memory that was first studied in ferromagnets (*30*). There is likely still a higher-order kind of memory arising from frustrated (i.e., antiferromagnetic) interactions between soft spots (*52*), although it has eluded experiments and molecular dynamics simulations to date. Last, the preceding kinds of memory are all rooted in a fixed population of soft spots, which seems inadequate to account for the anisotropy that encodes the direction of large deformations in our system and in bulk metallic glasses (*4*, *40*–*43*, *62*). Placed among the great variety of other systems with memory, a disordered solid is a chimera.

Our experiments suggest that considering amplitude variation and memory formation leads to an efficient, purely mechanical way to prepare disordered solids into a known state with reversible plasticity, minimal anisotropy and memory content, a maximal ability to form new memories, and a structural energy approaching the lowest mechanically accessible value. Some or all of these results might be generalized to other soft glassy systems, including artificial spin ice (*63*). In studying annealing, we have also clarified multiple ways a single glassy solid can remember an amplitude or direction and how each may be erased or avoided. One area where these insights could be valuable is rheometry of soft glassy solids, including concentrated emulsions (*5*, *7*): Besides the need to exclude memories of past deformations, one may also wish to suppress the transient response that follows each change in strain amplitude. To the extent that annealing can ensure a relaxed structure with return point memory behavior, the transients at the start of each test will be as short as possible, and the second cycle after a change in amplitude will be exactly like each one that follows (*46*, *52*).

Our results also show that in samples annealed by this method, neglecting interactions between soft spots, or more generally, between microscopic hysteretic subsystems, yields an unexpectedly successful microscopic model of rich plastic behaviors at strains up to several percent. With this approximation, we can catalog a sample’s population of these subsystems and then relate it to details of how that sample responds to amplitude variation and forms memories. We expect that our methods can be applied to probe and manipulate the non-equilibrium character of many other systems where one can measure microscopic change, including very different glassy matter such as crumpled sheets (*64*).

## MATERIALS AND METHODS

### Material samples

The material shown in Fig. 1A consists of polystyrene sulfate latex particles (Invitrogen), with diameters of 4.1 μm (Lot 1876103) and 5.4 μm (Lot 1818113) in roughly equal numbers. These particles are adsorbed at the interface between decane (“99%+,” ACROS Organics) and deionized water in a 60-mm-diameter glass dish (*11*). The particle suspension, with 50% ethanol as a spreading agent, is handled using pipette tips and Eppendorf tubes that are free of surface treatments (Axygen “Maxymum Recovery”).

The typical spacing between particle centers is *a* = 8.8 μm, corresponding to the first peak of the radial pair correlation function *g*(*r*) (*65*). In some movies, we observe voids and small aggregates that are presumably due to trace contaminants, but in experiments these act as rigid inclusions and have no special role in rearrangements or memory (see the Supplementary Materials).

Constant-amplitude data in Fig. 1D are from experiments described in (*26*), with a similar material, apparatus, and analysis methods as in this work; they are presented here for qualitative comparison.

### Area fraction measurement

We follow the procedure from (*26*). After applying a short-pass filter to remove background variations and cropping the image to remove boundary effects, we find the highest grayscale threshold that preserves small particles and the lowest grayscale threshold that does not merge neighboring particles. We fill holes in the resulting binary images and measure the fraction of dark pixels in each, which gives us a range of area fractions for our estimate.

### Apparatus

In our interfacial shear rheometer (ISR), the interface is pinned at the edges of two aluminum walls that form an open-ended channel 18 mm long and 2.4 mm wide. A 32-mm-long magnetized steel needle with a diameter of 233 μm is adsorbed at the interface in the channel. The needle is trapped at the center of the channel, parallel to the walls, by a pair of permanent magnets suspended above its ends, following the design of Tajuelo *et al.* (*37*) and related work by Qiao *et al.* (*66*). A translation stage (Physik Instrumente L-509 stage and C-884 controller) moves the magnets parallel to the channel, driving the needle and shearing the material between the needle and the walls. Compared with traditional ISRs that use stationary Helmholtz coils as a magnetic trap (*34*–*36*), it is easier to achieve strong field gradients that tightly couple the needle to the trap to better control strain for the present experiments. To minimize misalignment of the needle, the apparatus is oriented in the horizontal plane to match the local background magnetic field in the laboratory. In-house software and electronics control the motion and synchronize it with video capture.

### Shear deformation

We achieve a nominal strain amplitude γ_0_ by displacing the magnetic trap with amplitude γ_0_*R*, where *R* is the gap between the needle and each wall. Because of the elastic modulus of the material and the finite stiffness of the magnetic trap, the actual motion of the needle is slightly smaller than γ_0_*R*. We measure this actual strain by a least-squares fit of an affine transform to all particle motions and denote it γ_aff_.

When needed, the mapping from these discrete values to γ_aff_ is given by the extrema of strain during the readout protocol at the end of each experiment. In Fig. 4A, we also show γ_aff_ for comparison.

Shear is nearly quasistatic, in that the typical time scale for a rearrangement to complete (∼3 s) is much shorter than both the period of shearing (20 s) and the inverse of the maximum strain rate during memory experiments (64 s). Shear is also nearly uniform, but at that maximum strain rate, we detect slight nonuniformities because the interface is weakly coupled to bulk viscous flows in the oil and water, which have a different velocity profile (*36*). In the Supplementary Materials, we analyze this behavior and show that the effect is small: When the needle is moving the fastest (i.e., near γ = 0 when γ_0_ = 5%), the nonuniformity advances or retards the local shear strain by at most 0.2% strain relative to the global value, depending on location.

### Particle tracking

During deformation, we image ∼24,000 particles in an area that includes the needle and one wall. We use a long-distance microscope (Infinity K2/SC) and 4-megapixel machine-vision camera (Mikrotron 4CXP) at a magnification of 0.665 μm/pixel and a rate of 20 frames/s. High-throughput tracking is performed with the open-source “trackpy” software (*65*, *67*) using the channel-flow prediction and adaptive search features. An image registration algorithm assists tracking by measuring occasional global displacements of particles due to external vibrations of the microscope; subsequent analysis is insensitive to these global motions. To reduce the effect of spurious rearrangements caused by particle tracking errors, we discard any particle that is not tracked continuously over an entire set of samples, e.g., the entire readout process.

### Particle displacements

To find a particle’s displacement between two video frames for Fig. 1A and MSD measurements, we subtract the average motion of the surrounding material within radius *R*_disp_. This avoids spurious signals due to small motions of the camera or variation of the needle position, yielding $\Delta {\vec{r}}_{\text{local}}$ (*11*, *68*). We do not compute displacements of particles within *R*_disp_ of an edge of the field of view. To compute MSD, we use *R*_disp_ = 16.5*a*; in Fig. 1A, only we use *R*_disp_ = 12.5*a* to let us observe particles near the edge of the field of view. We found previously that choosing *R*_disp_ = 4.5*a* or 8.5*a* did not change our qualitative results (*26*).

### Identifying rearranging particles

While particle displacements allow us to observe rearranging soft spots and compute global differences, we also wish to identify the particles directly involved in a rearrangement, excluding its extended displacement field. We use the $D_{\text{min}}^{2}$ measure developed by Falk and Langer (*48*, *69*). Given particle positions at two instants, we consider each particle and its two “shells” of nearest neighbors (within radius of ∼2.5*a*) and find the best-fit affine transformation tensor that describes their displacements (*68*). $D_{\text{min}}^{2}$ is the mean-squared residual from this fit, normalized by *a*^2^. Figure 5B shows $D_{\text{min}}^{2}$ corresponding to the rearrangement in Fig. 1A.

In rare cases, particles with irregular shapes or sizes can be tracked but not located with sufficient consistency, so that a single particle may be briefly and erroneously displaced by 𝒪(1) px relative to its neighbors. The result is a solitary particle with a high value of $D_{\text{min}}^{2}$, very unlike the region of elevated $D_{\text{min}}^{2}$ that indicates an actual rearrangement of several particles. An effective denoising method is to remove each particle with $D_{\text{min}}^{2}$ greater than five times the median of its neighbors’ values (i.e. particles closer than 1.5*a*) and then recompute $D_{\text{min}}^{2}$ for all remaining particles. This procedure removes few particles in actual rearrangements. For example, only one such particle is missing from Fig. 5B.

## Supplementary Materials

### This PDF file includes:

Supplementary Text Figs. S1 to S9 References

## Notes

View/request a protocol for this paper from *Bio-protocol*.
