# Supplementary material for: Mechanical annealing and memories in a disordered solid
Source: Sci Adv. 2022 Oct 5;8(40):eabo1614. doi: 10.1126/sciadv.abo1614 (PMC9534499; doi:10.1126/sciadv.abo1614)
Supplement: Supplementary file 1 — Supplementary Text Figs. S1 to S9 References [file sciadv.abo1614_sm.pdf]

Science Advances
AAAS

Supplementary Materials for

Mechanical annealing and memories in a disordered solid

Nathan C. Keim and Dani Medina

Corresponding author: Nathan C. Keim, keim@psu.edu

Sci. Adv. **8**, eabo1614 (2022)
DOI: 10.1126/sciadv.abo1614

The PDF file includes:

Supplementary Text
Figs. S1 to S9
Legends for movies S1 to S3
References

Other Supplementary Material for this manuscript includes the following:

Movie S1 to S3

SUPPLEMENTARY TEXT AND FIGURES

Readout of 1 and 2 memories

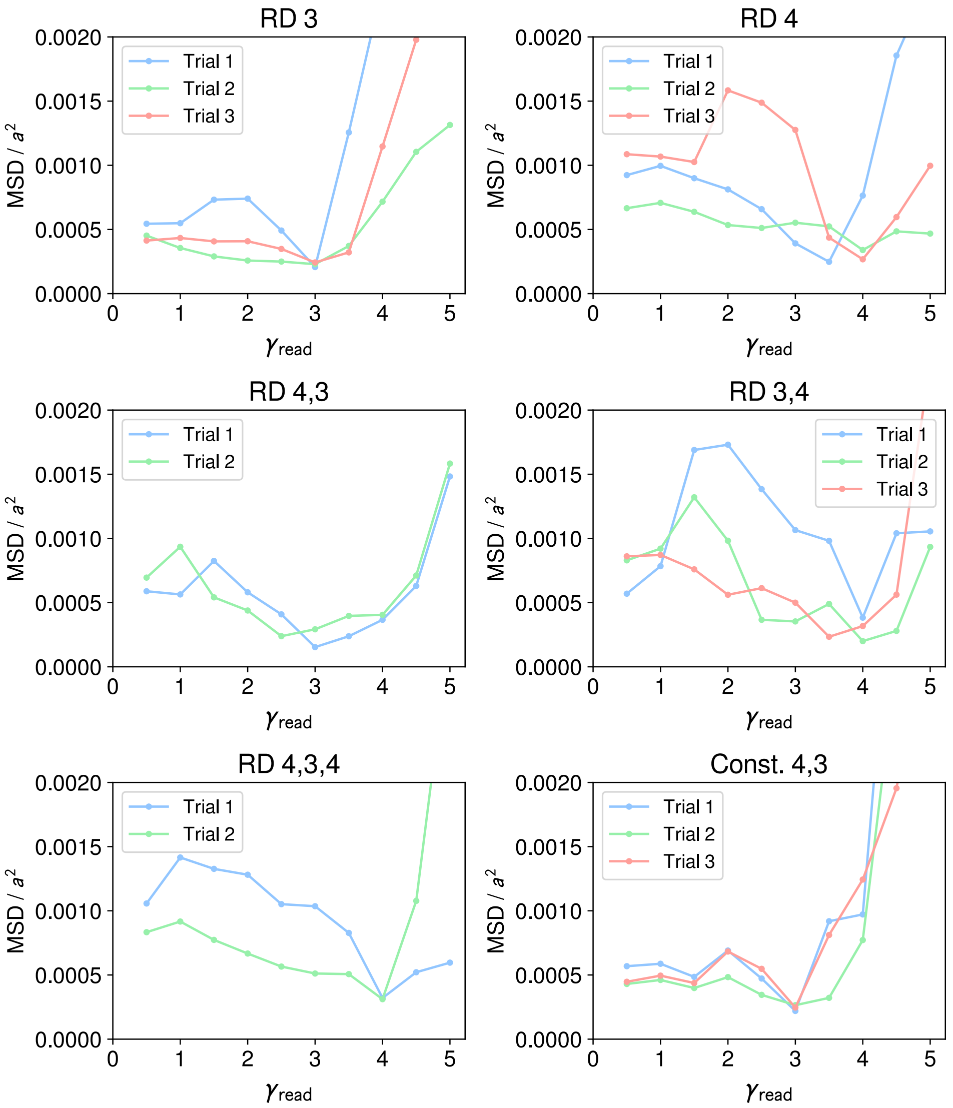

FIG. S1. **All trials used to generate the average curves in Figs. 1e, 3c, and 3d of the paper.** (The "No write" curve in Fig. 1e comes from the data in Fig. 4, as described in the paper.)

Figure S1 shows all of the individual readout curves that are averaged to make the single- and dual-memory readout curves in the paper. Each curve is consistent with the distinctive features of the average it contributes to, but the variation underscores the variability in soft spot populations that is examined in detail in Figs. 4–7 of the paper. There is also a tendency for some minima to occur at the sample to the left of the anticipated memory value, because of sporadic soft spots that unexpectedly rearrange (or fail to reverse). Typically the effect is subtle, but the most severe example of this is Trial 1 of the "RD 4" curve. To illustrate more typical readouts and put this worst case in context, Movies S1–S3 show rearrangement activity during each of the three "RD 4" trials.

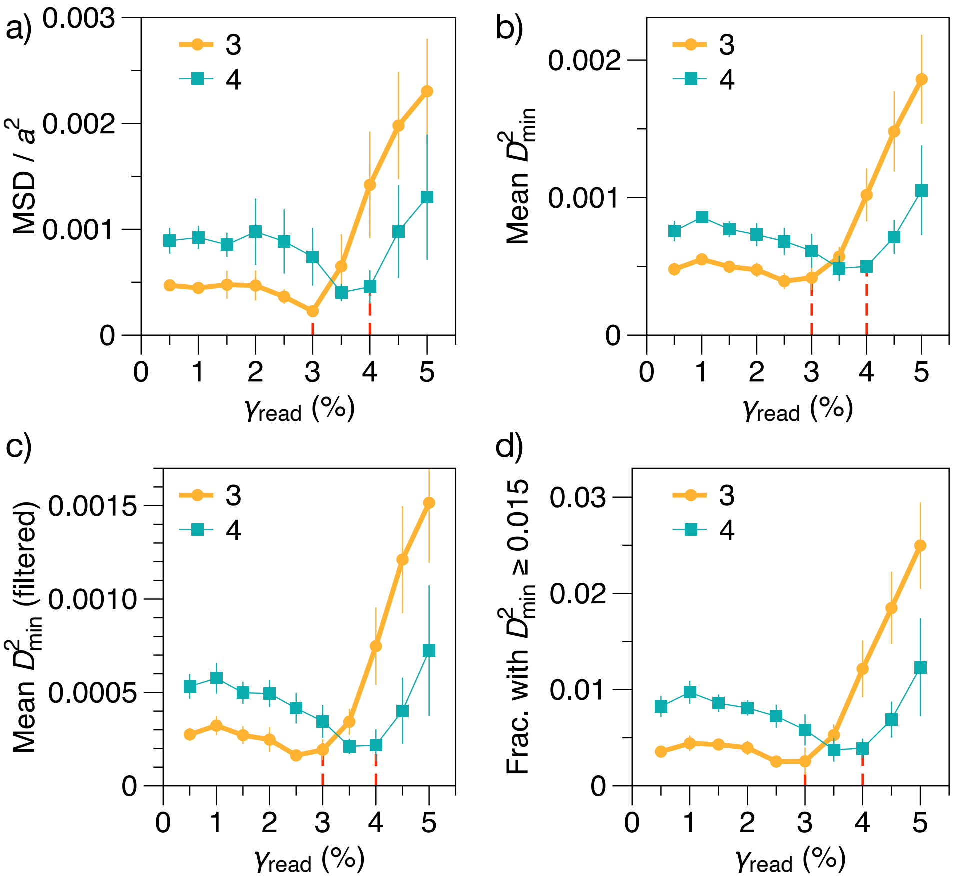

FIG. S2. **Comparison of metrics for reading single memories.** In the same experiments with memories written after ring-down (e.g. fig. 1e), four methods are used to measure differences in particle arrangements during read-out: **(a)** mean-squared displacement curves from fig. 1e of paper; **(b)** mean D^2_{min} of all particles; **(c)** mean D^2_{min}, but with ostensibly spurious single-particle rearrangements removed; **(d)** fraction of particles with $D^2_{\mathrm{min}} \geq 0.015$.

In principle, many different metrics for comparing states of the system can be used to read out memories. Figure S2 shows that besides mean squared displacement (MSD), one may also use metrics based on the non-affine displacement D^2_{min}. The Materials and Methods section of the paper describes the algorithms for computing local particle displacements as the basis for MSD; for computing the non-affine displacement D^2_{min}; and for filtering D^2_{min} to remove spurious single-particle rearrangements. In Fig. S2d we use the same $D^2_{\mathrm{min}} \geq 0.015$ threshold that we have used in previously-published analyses (*11, 26, 38*), and that we apply in Fig. 5 of the paper and discuss in the accompanying text. The choice of this threshold is further illustrated in Figs. 5(b, c) and Fig. S6. All of the methods shown in Fig. S2 are consistent with the expected memories and qualitatively consistent with each other, within error.

These results complement the image-subtraction results discussed below, wherein single memories may also be retrieved by comparing raw experimental images during readout. However our implementations of MSD and D^2_{min} are more suitable for these experiments than image subtraction, because they reject long-wavelength displacements of particles that are described below.

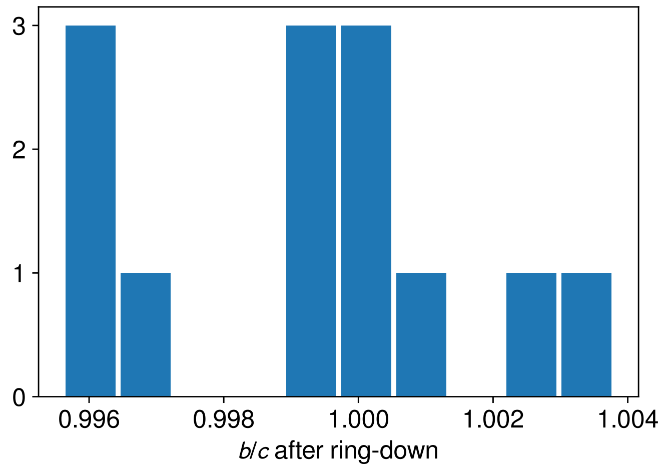

FIG. S3. **Distribution of nearest-neighbor asymmetry b/c (defined in Fig. 2 of main paper) after ring-down annealing.** In 13 trials, the mean is 0.9993 with standard deviation 0.0024.

Distribution of anisotropy after ring-down

In the text we analyze the pair correlation function $g(r, \theta)$ after various preparation protocols, and we measure its asymmetry as the ratio of ellipse radii b/c. We report the mean and standard deviation of b/c at $\gamma = 0$ after ring-down annealing, in 13 trials. Figure S3 shows the distribution of these values. It is notable that although we performed only two trials of annealing with constant 4% strain amplitude, their b/c values of 0.9988 and 0.9920 at $\gamma = 0$ suggest that they are likely drawn from a wider distribution than in Fig. S3.

Spatiotemporal structure of rearrangements

Figure S4 shows maps of $\gamma_i^{\pm}$ for Sample B, as Fig. 5(d, e) does for Sample A. Unlike in Sample A, a small number of particles did not return to the same positions at the end of the cycle; they are marked in green.

Figure S5 tests an additional property that is necessary for return-point memory: that rearrangements are hierarchical, meaning that there is a fixed order in which they may occur during forward shear, and another fixed order during reverse shear ("no passing") (*27, 31*). In practice, this means that the particles that may rearrange during a cycle with a given strain amplitude must be a subset of the particles that rearrange at larger amplitudes—smaller cycles are "nested" inside larger ones. We test this property during the ring-down sequence for Sample A (strain protocol shown in Fig. 4 of paper). For each cycle during ring-down, we obtain the maximal set of rearrangements by computing D^2_{min} between the extrema γ_0 and $-\gamma_0$ (as described in "Materials and Methods") and applying the threshold of 0.015. (Note that this is a superset of the soft spots that can be observed in two different states at $\gamma = 0$, which are shown in Fig. 5(c, d) of the paper.) In Fig. S5 we show the smallest amplitude that caused each particle to rearrange, $\gamma_{0,\text{min}}$. The expectation of hierarchy means that each particle should rearrange at all $\gamma_0 \geq \gamma_{0,\text{min}}$. Indeed, this is true for the vast majority (97%) of rearranging particles. However, the particles marked with red circles in the figure violate this condition. Most are isolated particles that could be attributed to noise or threshold effects. However, there are a few clusters of several particles each, along the periphery of larger soft spots. These clusters persist when the D^2_{min} threshold is doubled or halved (as in Fig. S6), which increases the likelihood that they are not artifacts of particle tracking or analysis. They may instead be due to mechanical noise, or

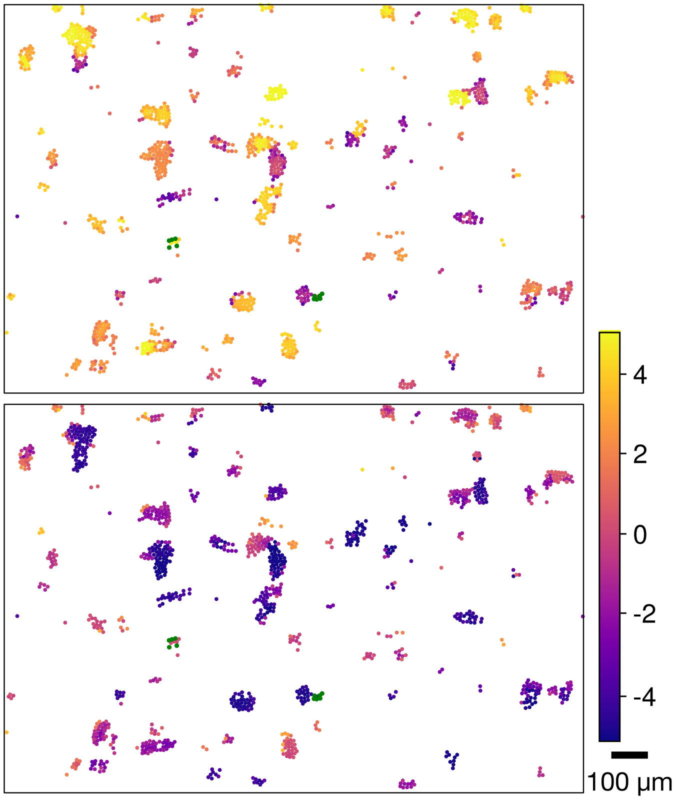

FIG. S4. **Spatiotemporal structure of rearrangements in Sample B in a single cycle.** Particles are colored by γ_i^+ and γ_i^-, as the counterpart to the Sample A results in Figs. 5(d, e). In this case several rearranging particles were discarded because their trajectories were not closed; they are marked in green.

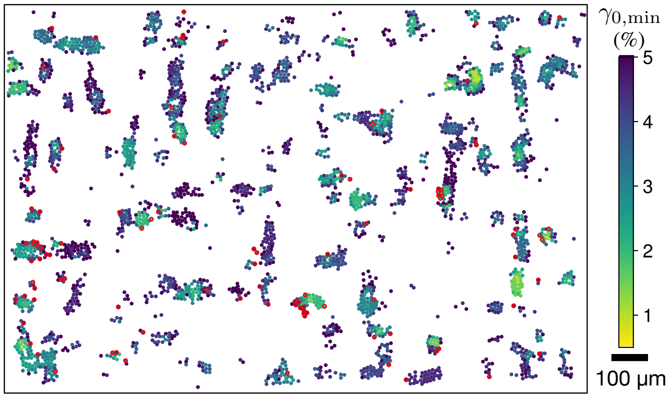

FIG. S5. **Test of whether the response of the system to variable-amplitude driving is hierarchical.** As amplitude is decreased, each cycle's rearrangement activity should be a subset of the previous cycle's. During ring-down in the experiment with Sample A (Fig. 4), each particle is colored by the smallest strain amplitude at which it rearranges. Red particles also had a *maximum* amplitude at which they were observed to rearrange, violating the expected hierarchy. Most violations are 1–2 particles, suggesting they are noise or artifacts of the threshold, but a few are extended regions at the periphery of larger soft spots.

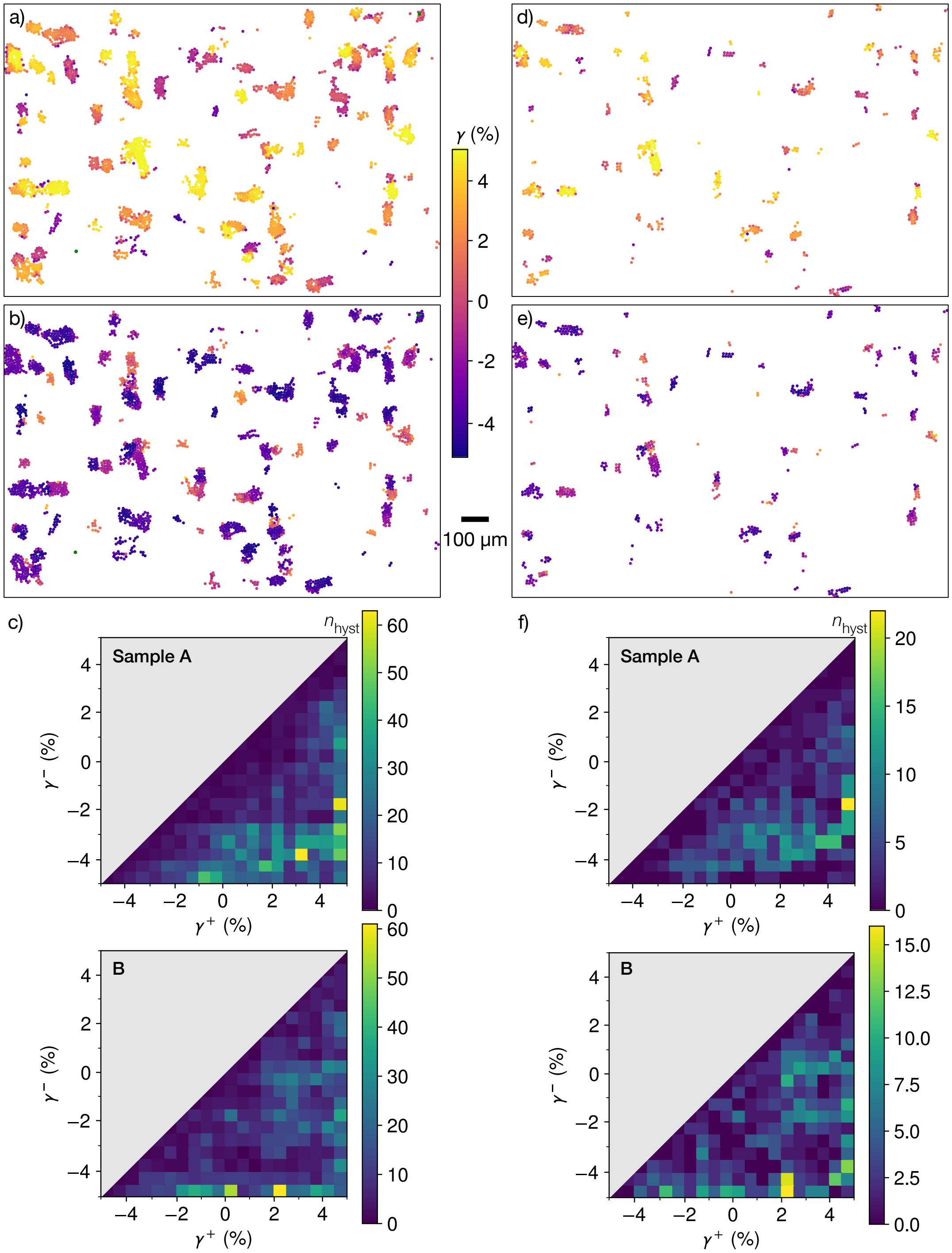

FIG. S6. **Spatiotemporal structure of rearrangements as in Figs. 5(d, e) and 6, for alternate values of the $D^2_{\rm min}$ threshold.** Analysis in the paper uses threshold 0.015, while **(a, b, c)** use the threshold 0.0075, and **(d, e, f)** use threshold 0.03. Upper panels show particles in Sample A colored by γ_i^+ (a, d) and γ_i^- (b, e), using the color bar between them. Lower panels (c, f) show histograms of $\gamma_i^\pm$ for each of Samples A and B, using the color bar to the right of each plot.

to frustrated interactions as mentioned in the paper.

Figure S6 explores the role of the D^2_{min} threshold used to determine whether a particle has participated in a rearrangement. In the text and in past analyses (*11, 38*) this is 0.015. Doubling or halving the threshold shrinks or grows the spatial extents of soft spots in our results as compared to Figs. 5(d, e), but it does not qualitatively change the distribution of strain thresholds $\gamma_i^{\pm}$ as compared to Fig. 6, so that these measurements remain consistent with each sample's response to amplitude variation (Fig. 4).

Nonuniform shear strain

We find that particles near the middle of the material (i.e. halfway between the needle and the wall) systematically lag behind the displacements that would be expected for uniform shear. This is due to the weak coupling of the interfacial material to the bulk (oil and water) viscous flow, which has a different velocity profile (*36, 70*). This lag is evident when comparing particle positions in two video frames that are ostensibly at the same value of global strain, but were taken during forward and reverse shear—as in the sampling protocol illustrated in Fig. 5a that was used to measure particles' $\gamma_i^{\pm}$.

To illustrate this effect, Fig. S7 compares particles' horizontal x positions between forward and reverse shear, averaged over 10 pairs of frames in the analysis of sample "B" near $\gamma = 0$, when the strain rate and the lag are largest. We plot half the difference, $\Delta x/2$, as a function of position y across the channel; the top of each image (near the fixed wall) is $y = 0$. Measurements are divided into 20 equally-spaced bins in y. Given the roughly uniform distribution of soft spots with respect to y, we would expect $\Delta x/2$ to average out to zero throughout the material, but instead there is a clear systematic difference, which is also evident in the image subtraction data described below. A positive $\Delta x/2$ means that a particle's x is larger during forward shear ($\dot{\gamma} > 0$), so that it lags behind the needle's motion (in the $-x$ direction for forward shear).

Examining the slopes in Fig. S7, we see that in these observations taken when the effect is largest, the local shear strain leads the global shear strain by $\sim$0.15% near the needle ($y \lesssim 500$ μm), and lags by $\sim$0.2% near the wall. This means that the values of $\gamma_i^{\pm}$ we report in Figs. 5 and 6 would be slightly different if they could be based on local shear strain at each soft spot. The net effect on our analysis of memory is small and subtle. The material's memory is defined in terms of global shear strain, and so in that sense, the $\gamma_i^{\pm}$ we measure are appropriate for this analysis and do not need to be corrected. Instead, the effect on our analysis comes from the fact that we drive the experiment at fixed frequency, and so decreasing γ_0 will lower the maximum strain rate and make the lag due to this viscous flow proportionally smaller—slightly and non-uniformly shifting the apparent $\gamma_i^{\pm}$ from the values we measured in the cycle with $\gamma_0 = 5\%$. However, even this is unlikely to affect our conclusions: the prominent differences between Samples A and B in Fig. 7g are robust to perturbing particles' $\gamma_i^{\pm}$ by $\sim \pm 0.2\%$.

Further studies are needed to systematically study the role of finite strain rate in this and other experiments. However, we note that the lagging effect we have discussed is an artifact of our experimental system's quasi-2D geometry (*36*), and not of the sampling and measurement methods we describe in the paper. In 3D materials, it should be possible to avoid this type of strain non-uniformity altogether.

Supporting data files

A URL to a permanent, downloadable archive of supporting data is provided in the paper. It contains image data sufficient to verify all of the qualitative results in the paper, in 4 directories:

- `asymmetry` has the raw images corresponding to the beginning of each of the curves in Fig. 2c, as well as from 12 additional ring-down movies. The mean and standard deviation of the asymmetry ratio b/c are reported in the text for these 13 frames taken from ring-down movies.

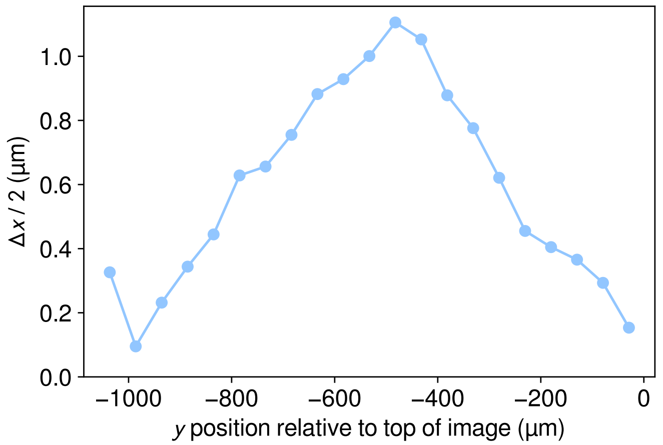

FIG. S7. **Profile of lagging displacements.** The horizontal x positions of particles are compared between forward and reverse shear within a single cycle, in pairs of frames with matched values of strain γ. Particles are binned according to y position, with $y = 0$ corresponding to the top of each video frame, near the fixed wall. 10 pairs of frames are averaged near $\gamma = 0$, when strain rate is fastest and the lag is largest. Plot shows the half-difference $\Delta x/2$. Uniform shear with no lag would correspond to $\Delta x = 0$; instead, particles lag behind the expected displacement, making the shear strain slightly nonuniform.

- `readout` has image subtractions from readout, corresponding to the MSD calculations for all readouts shown in Fig. 1e and Figs. 3(c, d).
- `nested` has image subtractions corresponding to all MSD calculations in Fig. 4b, for Samples A and B.
- `cycle` has image subtractions between each image during forward shear and its counterpart during reverse shear, corresponding to the D^2_{min} calculations used to obtain γ^+ and γ^- in Figs. 5(d, e) and Fig. S4, for samples A and B respectively. Files are labeled with the strain value of each pair.

Static structure images

Figure S8 shows an example image of static structure from the `asymmetry` directory, from which $g(r, \theta)$ may be calculated as in the paper. The horizontally-moving needle is at the bottom of each image, a fixed wall is at the top, and the magnification is 0.665 μm/pixel. Some frames in `asymmetry` show small aggregates that formed gradually over the 23-hour run of experiments due to the weakening of repulsive forces, presumably from the presence of trace surfactants (*71*). A smaller number of frames also have “voids”—regions 25–140 μm in size that are nearly devoid of particles, possibly due to a monolayer of adsorbed, insoluble contaminant.

Subtracted images

The subtracted images show that our qualitative results can be reproduced with this simple method, instead of particle tracking. Figure S9 shows two examples from the data archive, representing the recovery of a memory during readout, and the rearrangements caused by exceeding the constant annealing amplitude. To produce these subtracted images from a pair of raw images, we convert each raw image to floating-point

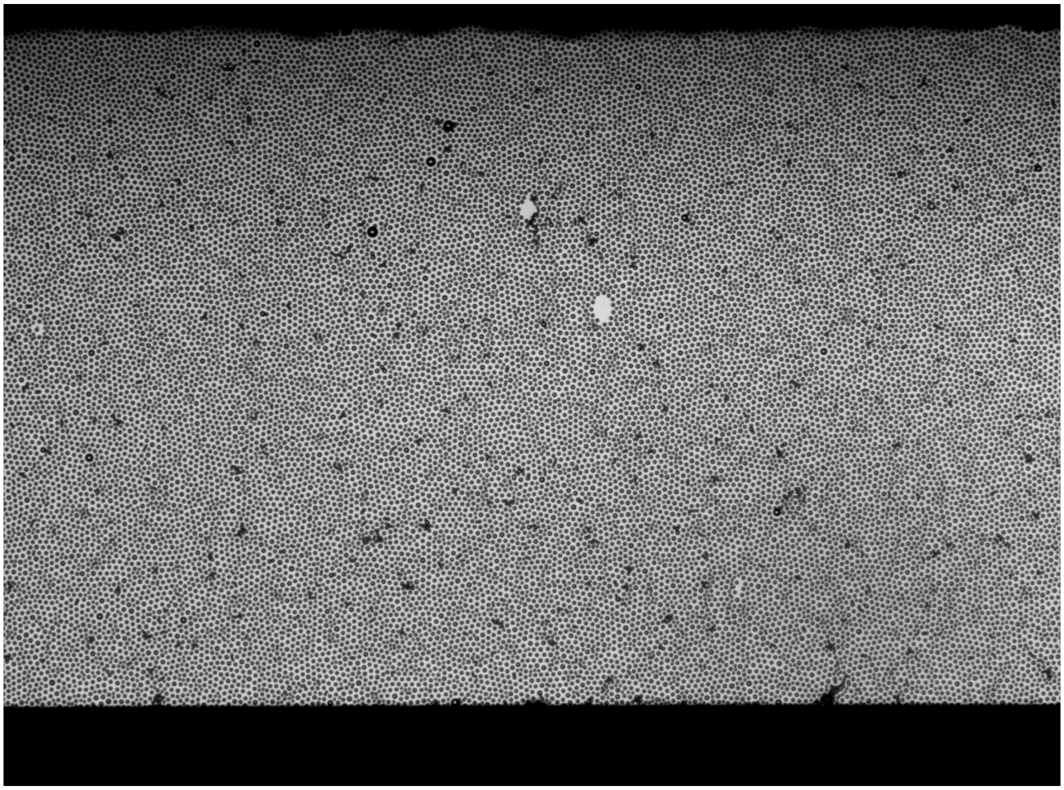

FIG. S8. **Example raw image of static structure prepared by ring-down annealing.** Width is 1550 μm. Fixed wall is at top; moving needle is at bottom. Sporadic small particle aggregates and two voids are visible.

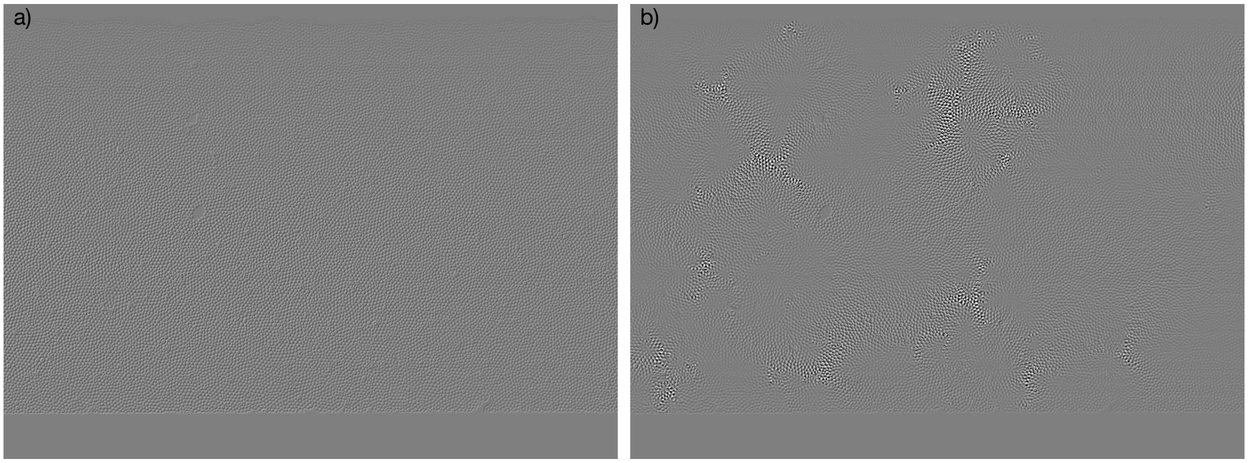

FIG. S9. **Representative images from memory readout after constant-amplitude annealing at 4% and writing of a memory of 3% strain. (a)** After a readout cycle with amplitude 3%, subtracting the image taken before readout reveals only small global displacements, indicating that the state before readout is recovered. **(b)** A readout cycle of amplitude 5% exceeds the annealing amplitude, resulting in several large new rearrangements. Width of each image is 1550 μm.

values between 0 and 1, subtract the earlier frame from the later one, and then clip the result to the range $[-0.5, 0.5]$ to make differences more visible; grey values in the resulting image files represent this range.

Soft spot rearrangements are apparent in images such as Fig. S9b as localized clusters of displaced particles, with an approximately quadrupolar morphology as in Fig. 1a (*72, 73*). Some images also show large-scale perturbations from mechanical noise that affects the needle and camera positions, and small mismatches in strain due to the finite frame rate. These are all rejected by the quantitative methods used in the paper and described in its Materials and Methods section, which use the displacement of each particle relative to nearby particles. Taken together, these images also show that the aggregates and voids act as rigid inclusions and do not appear to play any special role in rearrangements or memory—for example, the two small voids in the material in Fig. S9 that are visible only because of a small global displacement.

The subtracted images in the `cycle` directory, based on the sampling protocol illustrated in Fig. 5a, deserve additional comment. Because we always subtract the earlier frame from the later one, the displacements switch signs near $\gamma = 0$, when we switch from making comparisons in the first half of the sampled cycle to the second half. Additionally, these images show the slight large-scale nonuniformity of the shear deformation discussed above, and confirm that the effect disappears as the needle slows down near the extrema of strain.

SUPPLEMENTARY MOVIES

Movie S1. Readout of a memory of 4% strain amplitude, for Trial 1 of 3. These trials make up the "4" curve in Fig. 1e and the "RD 4" curve in Fig. 3(c, d); they are plotted separately in Fig. S1. The upper panel shows all particles colored by D^2_{min}, as computed between the markers on the strain-vs.-time plot in the lower panel. Frames correspond to one of every 10 video frames in the original experiment (i.e., 40 per cycle), so that the frame at the end of each cycle in these movies may not show $\gamma = 0$ exactly. The configuration after writing is recovered (overall D^2_{min} minimized) after the memory amplitude is applied again. In this trial only, one large errant soft spot remains after the memory amplitude is reapplied. We plot affine strain as defined in Materials and Methods. The moving needle is at the bottom of the plotted region, a fixed wall is at the top, and the height of the plotted region is 1.50 mm.

Movie S2. Readout of a memory of 4% strain amplitude, for Trial 2 of 3. These trials make up the "4" curve in Fig. 1e and the "RD 4" curve in Fig. 3(c, d); they are plotted separately in Fig. S1. The upper panel shows all particles colored by D^2_{min}, computed between the markers on the strain-vs.-time plot in the lower panel. Frames correspond to one of every 10 video frames in the original experiment (i.e., 40 per cycle), so that the frame at the end of each cycle in these movies may not show $\gamma = 0$ exactly. The configuration after writing is recovered (overall D^2_{min} minimized) after the memory amplitude is applied again. We plot affine strain as defined in Materials and Methods. The moving needle is at the bottom of the plotted region, a fixed wall is at the top, and the height of the plotted region is 1.50 mm.

Movie S3. Readout of a memory of 4% strain amplitude, for Trial 3 of 3. These trials make up the "4" curve in Fig. 1e and the "RD 4" curve in Fig. 3(c, d); they are plotted separately in Fig. S1. The upper panel shows all particles colored by D^2_{min}, computed between the markers on the strain-vs.-time plot in the lower panel. Frames correspond to one of every 10 video frames in the original experiment (i.e., 40 per cycle), so that the frame at the end of each cycle in these movies may not show $\gamma = 0$ exactly. The configuration after writing is recovered (overall D^2_{min} minimized) after the memory amplitude is applied again. We plot affine strain as defined in Materials and Methods. The moving needle is at the bottom of the plotted region, a fixed wall is at the top, and the height of the plotted region is 1.50 mm.

REFERENCES AND NOTES

1. B. Verlinden, J. Driver, I. Samajdar, R. D. Doherty, *Thermo-Mechanical Processing of Metallic Materials* (Elsevier, 2007).

2. J. Schroers, Bulk metallic glasses. *Phys. Today* **66**, 32–37 (2013).

3. A. L. Greer, Y. Q. Cheng, E. Ma, Shear bands in metallic glasses. *Mater. Sci. Eng. R Rep.* **74**, 71–132 (2013).

4. Y. Sun, A. Concustell, A. L. Greer, Thermomechanical processing of metallic glasses: Extending the range of the glassy state. *Nat. Rev. Mater.* **1**, 16039 (2016).

5. C. W. Macosko, *Rheology* (Wiley-VCH, 1994).

6. R. G. Larson, *The Structure and Rheology of Complex Fluids* (Oxford Univ. Press, 1998).

7. H. S. Kim, T. G. Mason, Advances and challenges in the rheology of concentrated emulsions and nanoemulsions. *Adv. Colloid Interface Sci.* **247**, 397–412 (2017).

8. E. D. Cubuk, R. J. S. Ivancic, S. S. Schoenholz, D. J. Strickland, A. Basu, Z. S. Davidson, J. Fontaine, J. L. Hor, Y. R. Huang, Y. Jiang, N. C. Keim, K. D. Koshigan, J. A. Lefever, T. Liu, X. G. Ma, D. J. Magagnosc, E. Morrow, C. P. Ortiz, J. M. Rieser, A. Shavit, T. Still, Y. Xu, Y. Zhang, K. N. Nordstrom, P. E. Arratia, R. W. Carpick, D. J. Durian, Z. Fakhraai, D. J. Jerolmack, D. Lee, J. Li, R. Riggleman, K. T. Turner, A. G. Yodh, D. S. Gianola, A. J. Liu, Structure-property relationships from universal signatures of plasticity in disordered solids. *Science* **358**, 1033–1037 (2017).

9. I. Regev, T. Lookman, C. Reichhardt, Onset of irreversibility and chaos in amorphous solids under periodic shear. *Phys. Rev. E* **88**, 062401 (2013).

10. N. C. Keim, P. E. Arratia, Yielding and microstructure in a 2d jammed material under shear deformation. *Soft Matter* **9**, 6222 (2013).

11. N. C. Keim, P. E. Arratia, Mechanical and microscopic properties of the reversible plastic regime in a 2d jammed material. *Phys. Rev. Lett.* **112**, 028302 (2014).

12. J. R. Royer, P. M. Chaikin, Precisely cyclic sand: Self-organization of periodically sheared frictional grains. *Proc. Natl. Acad. Sci. U.S.A.* **112**, 49–53 (2015).

13. N. V. Priezjev, Heterogeneous relaxation dynamics in amorphous materials under cyclic loading. *Phys. Rev. E* **87**, 052302 (2013).

14. D. Fiocco, G. Foffi, S. Sastry, Encoding of memory in sheared amorphous solids. *Phys. Rev. Lett.* **112**, 025702 (2014).

15. K. H. Nagamanasa, S. Gokhale, A. K. Sood, R. Ganapathy, Experimental signatures of a nonequilibrium phase transition governing the yielding of a soft glass. *Phys. Rev. E* **89**, 062308 (2014).

16. P. Das, A. D. S. Parmar, S. Sastry, Annealing glasses by cyclic shear deformation. arXiv:1805.12476 [cond-mat.soft] (2018).

17. E. Schinasi-Lemberg, I. Regev, Annealing and rejuvenation in a two-dimensional model amorphous solid under oscillatory shear. *Phys. Rev. E* **101**, 012603 (2020).

18. W. Yeh, M. Ozawa, K. Miyazaki, T. Kawasaki, L. Berthier, Glass stability changes the nature of yielding under oscillatory shear. *Phys. Rev. Lett.* **124**, 225502 (2020).

19. N. V. Priezjev, Accessing a broader range of energy states in metallic glasses by variable-amplitude oscillatory shear. arXiv:2101.03638v1 [cond-mat.soft] (23 February 2021).

20. H. Bhaumik, G. Foffi, S. Sastry, The role of annealing in determining the yielding behavior of glasses under cyclic shear deformation. *Proc. Natl. Acad. Sci. U.S.A.* **118**, e2100227118 (2021).

21. S. Sastry, Models for the yielding behavior of amorphous solids. *Phys. Rev. Lett.* **126**, 255501 (2021).

22. C. Liu, E. E. Ferrero, E. A. Jagla, K. Martens, A. Rosso, L. Talon, The fate of shear-oscillated amorphous solids. arXiv:2012.15310v3 [cond-mat.soft] (30 December 2020).

23. M. Lundberg, K. Krishan, N. Xu, C. S. O’Hern, M. Dennin, Reversible plastic events in amorphous materials. *Phys. Rev. E* **77**, 041505 (2008).

24. I. Regev, J. Weber, C. Reichhardt, K. A. Dahmen, T. Lookman, Reversibility and criticality in amorphous solids. *Nat. Comms.* **6**, 8805–8808 (2015).

25. M. Adhikari, S. Sastry, Memory formation in cyclically deformed amorphous solids and sphere assemblies. *Eur. Phys. J. E* **41**, 105 (2018).

26. N. C. Keim, J. Hass, B. Kroger, D. Wieker, Global memory from local hysteresis in an amorphous solid. *Phys. Rev. Res.* **2**, 012004 (2020).

27. M. Mungan, S. Sastry, K. Dahmen, I. Regev, Networks and hierarchies: How amorphous materials learn to remember. *Phys. Rev. Lett.* **123**, 178002 (2019).

28. I. Regev, I. Attia, K. Dahmen, S. Sastry, M. Mungan, The topology of the energy landscape of sheared amorphous solids and the irreversibility transition. arXiv:2101.01083v1 [cond-mat.soft] (4 January 2021).

29. S. Mukherji, N. Kandula, A. K. Sood, R. Ganapathy, Strength of mechanical memories is maximal at the yield point of a soft glass. *Phys. Rev. Lett.* **122**, 158001 (2019).

30. J. Barker, D. Schreiber, B. Huth, D. H. Everett, Magnetic hysteresis and minor loops: Models and experiments. *Proc. R. Soc. Lond. A* **386**, 251–261 (1983).

31. J. P. Sethna, K. Dahmen, S. Kartha, J. A. Krumhansl, B. W. Roberts, J. D. Shore, Hysteresis and hierarchies: Dynamics of disorder-driven first-order phase transformations. *Phys. Rev. Lett.* **70**, 3347–3350 (1993).

32. N. C. Keim, J. D. Paulsen, Z. Zeravcic, S. Sastry, S. R. Nagel, Memory formation in matter. *Rev. Mod. Phys.* **91**, 035002 (2019).

33. K. Masschaele, B. J. Park, E. M. Furst, J. Fransaer, J. Vermant, Finite ion-size effects dominate the interaction between charged colloidal particles at an oil-water interface. *Phys. Rev. Lett.* **105**, 048303 (2010).

34. G. T. J. Shahin, "Stress deformation interfacial rheometer," thesis, University of Pennsylvania, Philadelphia, PA (1986).

35. C. F. Brooks, G. G. Fuller, C. W. Frank, C. R. Robertson, An interfacial stress rheometer to study rheological transitions in monolayers at the air-water interface. *Langmuir* **15**, 2450–2459 (1999).

36. S. Reynaert, C. F. Brooks, P. Moldenaers, J. Vermant, G. G. Fuller, Analysis of the magnetic rod interfacial stress rheometer. *J. Rheol.* **52**, 261–285 (2008).

37. J. Tajuelo, J. M. Pastor, M. A. Rubio, A magnetic rod interfacial shear rheometer driven by a mobile magnetic trap. *J. Rheol.* **60**, 1095–1113 (2016).

38. N. C. Keim, P. E. Arratia, Role of disorder in finite-amplitude shear of a 2d jammed material. *Soft Matter* **11**, 1539–1546 (2015).

39. K. L. Galloway, X. Ma, N. C. Keim, D. J. Jerolmack, A. G. Yodh, P. E. Arratia, Scaling of relaxation and excess entropy in plastically deformed amorphous solids. *Proc. Natl. Acad. Sci. U.S.A.* **117**, 11887–11893 (2020).

40. K. L. Galloway, E. G. Teich, X.-g. Ma, C. Kammer, I. R. Graham, N. C. Keim, C. Reina, D. J. Jerolmack, A. G. Yodh, P. E. Arratia, Relationships among structure, memory, and flow in sheared disordered materials. arXiv:2105.06610v1 [cond-mat.soft] (16 March 2021).

41. R. Ott, M. Heggen, M. Feuerbacher, E. Park, D. Kim, M. Kramer, M. Besser, D. Sordelet, Anelastic strain and structural anisotropy in homogeneously deformed cu64.5zr35.5 metallic glass. *Acta Mater.* **56**, 5575–5583 (2008).

42. Y. Sun, A. Concustell, M. Carpenter, J. Qiao, A. Rayment, A. Greer, Flow-induced elastic anisotropy of metallic glasses. *Acta Mater.* **112**, 132–140 (2016).

43. E. Teich, K. Galloway, P. Arratia, D. Bassett, Crystalline shielding mitigates structural rearrangement and localizes memory in jammed systems under oscillatory shear. *Sci. Adv.* **7**, eabe3392 (2021).

44. J. D. Paulsen, N. C. Keim, Minimal descriptions of cyclic memories. *Proc. R. Soc. Lond. A* **475**, 20180874 (2019).

45. O. Perković, J. P. Sethna, Improved magnetic information storage using return-point memory. *J. Appl. Phys.* **81**, 1590 (1997).

46. N. C. Keim, S. R. Nagel, Generic transient memory formation in disordered systems with noise. *Phys. Rev. Lett.* **107**, 010603 (2011).

47. J. D. Paulsen, N. C. Keim, S. R. Nagel, Multiple transient memories in experiments on sheared non-brownian suspensions. *Phys. Rev. Lett.* **113**, 068301 (2014).

48. M. L. Falk, J. S. Langer, Deformation and failure of amorphous, solidlike materials. *Annu. Rev. Condens. Matter Phys.* **2**, 353–373 (2011).

49. M. L. Manning, A. J. Liu, Vibrational modes identify soft spots in a sheared disordered packing. *Phys. Rev. Lett.* **107**, 108302 (2011).

50. N. Perchikov, E. Bouchbinder, Variable-amplitude oscillatory shear response of amorphous materials. *Phys. Rev. E* **89**, 062307 (2014).

51. N. Keim, J. Paulsen, Multiperiodic orbits from interacting soft spots in cyclically sheared amorphous solids. *Sci. Adv.* **7**, eabg7685 (2021).

52. C. Lindeman, S. Nagel, Multiple memory formation in glassy landscapes. *Sci. Adv.* **7**, eabg7133 (2021).

53. F. Preisach, Über die magnetische Nachwirkung. *Z. Physik* **94**, 277–302 (1935).

54. A. Szulc, M. Mungan, I. Regev, Cooperative effects driving the multi-periodic dynamics of cyclically sheared amorphous solids. arXiv:2111.14155v1 [cond-mat.soft] (28 November 2021).

55. A. Szulc, O. Gat, I. Regev, Forced deterministic dynamics on a random energy landscape: Implications for the physics of amorphous solids. *Phys. Rev. E* **101**, 052616 (2020).

56. M. O. Lavrentovich, A. J. Liu, S. R. Nagel, Period proliferation in periodic states in cyclically sheared jammed solids. *Phys. Rev. E* **96**, 020101 (2017).

57. K. Khirallah, B. Tyukodi, D. Vandembroucq, C. E. Maloney, Yielding in an integer automaton model for amorphous solids under cyclic shear. *Phys. Rev. Lett.* **126**, 218005 (2021).

58. E. Nowak, J. Knight, M. Povinelli, H. Jaeger, S. Nagel, Reversibility and irreversibility in the packing of vibrated granular material. *Powder Technol.* **94**, 79–83 (1997).

59. M. Nicolas, P. Duru, O. Pouliquen, Compaction of a granular material under cyclic shear. *Eur. Phys. J. E* **3**, 309–314 (2000).

60. Z. A. Benson, A. Peshkov, D. C. Richardson, W. Losert, Memory in 3D cyclically driven granular material. arXiv:2010.04150v1 [cond-mat.soft] (8 October 2020).

61. F. Arceri, E. I. Corwin, V. F. Hagh, Marginal stability in memory training of jammed solids. *Phys. Rev. E* **104**, 044907 (2021).

62. S. Patinet, A. Barbot, M. Lerbinger, D. Vandembroucq, A. Lemaître, Origin of the bauschinger effect in amorphous solids. *Phys. Rev. Lett.* **124**, 205503 (2020).

63. R. F. Wang, J. Li, W. McConville, C. Nisoli, X. Ke, J. W. Freeland, V. Rose, M. Grimsditch, P. Lammert, V. H. Crespi, P. Schiffer, Demagnetization protocols for frustrated interacting nanomagnet arrays. *J. Appl. Phys.* **101**, 09J104 (2007).

64. D. Shohat, D. Hexner, Y. Lahini, Memory from coupled instabilities in unfolded crumpled sheets. *Proc. Natl. Acad. Sci. U.S.A.* **119**, e2200028119 (2022).

65. D. B. Allan, T. Caswell, N. C. Keim, C. M. van der Wel, Trackpy v0.4.2, DOI:10.5281/zenodo.3492186 (2019).

66. Y. Qiao, C. Fan, Z. Liu, D. Medina, N. C. Keim, X. Cheng, Miniature magnetic rod interfacial stress rheometer for general-purpose microscopes. *J. Rheol.* **65**, 1103–1110 (2021).

67. J. C. Crocker, D. G. Grier, Methods of digital video microscopy for colloidal studies. *J. Colloid Interf. Sci.* **179**, 298–310 (1996).

68. N. C. Keim, Philatracks v0.2, 10.5281/zenodo.11459 (2014).

69. M. L. Falk, J. S. Langer, Dynamics of viscoplastic deformation in amorphous solids. *Phys. Rev. E* **57**, 7192–7205 (1998).

70. T. Verwijlen, P. Moldenaers, H. A. Stone, J. Vermant, Study of the flow field in the magnetic rod interfacial stress rheometer. *Langmuir* **27**, 9345–9358 (2011).

71. B. J. Park, J. P. Pantina, E. M. Furst, M. Oettel, S. Reynaert, J. Vermant, Direct measurements of the effects of salt and surfactant on interaction forces between colloidal particles at water-oil interfaces. *Langmuir* **24**, 1686–1694 (2008).

72. J. D. Eshelby, The determination of the elastic field of an ellipsoidal inclusion, and related problems. *Proc. R. Soc. Lond. A* **241**, 376–396 (1957).

73. G. Picard, A. Ajdari, F. Lequeux, L. Bocquet, Elastic consequences of a single plastic event: A step towards the microscopic modeling of the flow of yield stress fluids. *Eur. Phys. J. E* **15**, 371–381 (2004).
